# Harnessing demographic differences in organizations: What moderates the effects of workplace diversity?

**DOI:** 10.1002/job.2040

**Published:** 2015-07-22

**Authors:** Yves R.F. Guillaume, Jeremy F. Dawson, Lilian Otaye‐Ebede, Stephen A. Woods, Michael A. West

**Affiliations:** ^1^ Aston Business SchoolAston UniversityBirminghamU.K.; ^2^ Sheffield University Management SchoolUniversity of SheffieldSheffieldU.K.; ^3^ Liverpool Business SchoolLiverpool John Moores UniversityLiverpoolU.K.; ^4^ Surrey Business SchoolUniversity of SurreyGuildfordU.K.; ^5^ Lancaster University Management SchoolLancaster UniversityLancasterU.K.

**Keywords:** relational demography, work group diversity, organizational diversity, TMT diversity, workplace demography, diversity management

## Abstract

To account for the double‐edged nature of demographic workplace diversity (i.e,. relational demography, work group diversity, and organizational diversity) effects on social integration, performance, and well‐being‐related variables, research has moved away from simple main effect approaches and started examining variables that moderate these effects. While there is no shortage of primary studies of the conditions under which diversity leads to positive or negative outcomes, it remains unclear which contingency factors make it work. Using the Categorization‐Elaboration Model as our theoretical lens, we review variables moderating the effects of workplace diversity on social integration, performance, and well‐being outcomes, focusing on factors that organizations and managers have control over (i.e., strategy, unit design, human resource, leadership, climate/culture, and individual differences). We point out avenues for future research and conclude with practical implications. © 2015 The Authors. Journal of Organizational Behavior published by John Wiley & Sons, Ltd

The business case for diversity holds that when workplace diversity (i.e., relational demography, work group diversity, and organizational diversity) works, it benefits the organization through more innovations, better decision‐making, a larger talent pool, and a wider customer base (Cox, 1993). However, diversity does not always work, being linked to lower employee morale (Tsui, Egan, & O'Reilly, 1992), more conflicts (Jehn, Northcraft, & Neale, 1999), and poorer job performance (Chatman, Polzer, Barsade, & Neale, 1998). To account for this double‐edged nature of diversity, research has moved away from simple main effect approaches and started investigating variables that moderate the effects of diversity on social integration, well‐being, and performance related variables (cf. Joshi, Liao, & Roh, 2011; van Knippenberg & Schippers, 2007). While there is no shortage of primary studies of the conditions under which diversity leads to positive or negative outcomes, it remains unclear which contingency factors make it work (Guillaume, Dawson, Woods, Sacramento, & West, 2013). In the literature, there are many examples of predicted moderators for which empirical support was not found, often leaving managers and organizations perplexed about how to manage diversity effectively (Avery & McKay, 2010).

To clarify the issue, meta‐analytic reviews (e.g., Guillaume, Brodbeck, & Riketta, 2012; Joshi & Roh, 2009; Thatcher & Patel, 2011; van Dijk, van Engen, & van Knippenberg, 2012) are of limited use because they are bound by their methodology to the investigation of contextual and methodological moderators derived from study characteristics (e.g., industry setting, study setting, diversity type, diversity operationalization, team type, team longevity, team interdependence, team size, and task type). They can say very little about moderator variables examined within studies (e.g., diversity climate, transformational leadership, or diversity beliefs) because the coefficients of interaction terms are not generally comparable between studies because of differing study design (Peterson & Brown, 2005), and conditional effects are often difficult to derive and interpret based on these metrics (cf. Kristof‐Brown, Zimmerman, & Johnson, 2005). Similarly, earlier qualitative reviews either did not look at moderating variables (e.g., Joshi et al., 2011) or were restricted by the number of primary studies available (e.g., van Knippenberg & Schippers, 2007). No review is available that evaluates what management practices moderate the effects of diversity (cf. Avery & McKay, 2010). The aim of our paper is therefore to take stock and provide a comprehensive and up‐to‐date qualitative review of variables moderating the effects of diversity on social integration, performance, and well‐being‐related variables, focusing on factors that organizations and managers have control over. In the following, we outline the scope of our review, define key variables, review the literature on what moderates diversity effects, discuss the implications of our findings for theory and practice, and point out future directions for research in this arena.

## Scope of the Review

In line with research in workplace demography (Joshi et al., 2011), we apply the term workplace diversity to any form that relational demography (i.e., individual level dissimilarity from peers), work group diversity, and organizational diversity may take (e.g., separation, variety, and disparity, Harrison & Klein, 2007; actual and perceived, Harrison, Price, Gavin, & Florey, 2002; faultlines, Lau & Murnighan, 1998). We subsume research in top management team (TMT) diversity under organizational diversity because of its focus on organizational level outcomes (cf. Joshi et al., 2011). Our review focuses on demographic attributes, such as gender, race/ethnicity, age, tenure, education, and functional background, because of their relevance and importance to research and organizations (cf. Williams & O'Reilly, 1998). Grounded in the social categorization/similarity attraction perspective and the information/decision‐making perspective, research in workplace diversity has mainly looked at two broad sets of outcome variables: social integration variables (e.g., conflict, cohesion, and attachment) and performance‐related variables (e.g., organizational performance, work group performance and innovation, and individual in‐role and extra‐role performances; cf. Joshi et al., 2011). More recently, research in workplace diversity building on the social categorization perspective has also started examining employee's well‐being‐related variables, such as stress and health (e.g., Wegge, Roth, Kanfer, Neubach, & Schmidt, 2008). Accordingly, our review investigates what moderates the effects of workplace diversity on performance, social integration, and employee well‐being variables.

To access the relevant literature, we conducted a manual search of the latest qualitative and quantitative reviews on work group and organizational diversity (Joshi et al., 2011; Joshi & Roh, 2009; Thatcher & Patel, 2011; van Dijk et al., 2012; van Knippenberg & Schippers, 2007; Williams & O'Reilly, 1998) as well as on relational demography (Guillaume et al., 2012; Joshi et al., 2011). To identify further studies, we searched relevant databases (ISIWeb of Knowledge, PsycInfo, and ABI/INFORM) for empirical quantitative studies that looked at the moderated effects of workplace diversity (i.e., relational demography, work group diversity, and organizational diversity) on social integration, performance, and health‐related outcomes and were published or in press in peer reviewed journals. We included studies that examined higher‐order interactions and studies that treated workplace diversity variables as a moderator rather than an independent variable, as long as the results could be re‐interpreted as a moderated workplace diversity effect. We also discuss the findings of previous meta‐analyses if relevant. As our review is intended to inform organizations and managers about how to manage workplace diversity effectively, we only included studies that examined moderating variables over which organizations and managers have control. While analysis of the papers that we found did not reveal any obvious structure into which the moderators fell, we tried to organize them into substantial themes that occur in the diversity management literature (Avery & McKay, 2010; Guillaume, Dawson, et al., 2014). Accordingly, we categorized moderating variables into the following groups: strategy, unit design, human resource (HR) practices, climate and culture, leadership, and individual differences.

Based on Chandler's seminal work, we define *strategy* as “the determination of the basic long‐term goals and objectives of an enterprise, and the adoption of courses of action and the allocation of resources necessary for carrying out these goals” (1962, p. 13).

Because a fit between environmental conditions and organizational capabilities/resources is critical to organizational performance (cf. Richard, 2000), we have also subsumed studies under this category, which examined environmental characteristics, such as change, instability, uncertainty, complexity, and customer demographic diversity. In line with Cohen and Bailey, we define *unit design* as “those features of the task, group, and organization that can be directly manipulated by managers to create the conditions for effective performance” (1997, p. 243) and differentiate between composition design (e.g., size, demographics, type of diversity, and faultline strength) and task design variables (e.g., task characteristics, type of team, interdependence, collocation, autonomy, empowerment, decentralization, meeting informality, and decision support systems). As effective unit design has been found to be contingent on lifecycle/temporal variables, such as group member tenure, team longevity, and time (cf. Hackman & Wageman, 2005), we also subsumed these variables under unit design. Based on the HR management literature, we refer to *HR practices* as a set of internally consistent policies and practices, such as staffing, training, appraisals, rewards, and promotions, designed and implemented to ensure that a firm's human capital contribute to the achievement of its business objectives (Delery & Doty, 1996). We adopt the broad definitions of *climate* and *culture* given by Schneider and Barbera (2014), who refer to climate as the meaning employees attach to the policies, practices, and procedures and the behaviors that get rewarded, supported, and expected at work; whereas culture is the meaning employees derive about the basic assumptions, values, and beliefs that underlie their experiences at work. As climate has been conceptualized both in terms of aggregated scores and individual perceptions (Ostroff, Kinicki, & Muhammad, 2012), we include both unit‐level measurements and individual‐level measurements of climate in this category. We define *leadership* as “the ability of an individual to influence, motivate, and enable others to contribute toward the effectiveness and success of the organizations of which they are members” (House, Hanges, Javidan, Dorfman, & Gupta, 2004, p. 15) and include studies in this category that looked at leader characteristics, leadership styles, or leader–follower relationship. Further, we refer to *individual differences* as generalized attitudes, motivational or cognitive styles, and personality traits. We also included cognitive ability and skills in this category. These variables are therefore differentiated from attitudes or perceptions that are linked to a specific workplace feature (e.g., climate perceptions).

Although climate is often understood to include perceptions of HR practices and strategy, we kept it separate from actual strategy and actual HR practices because climate, strategy, and HR practices are often thought to be conceptually distinct and influenced by different people in an organization (Ostroff et al., 2012). Strategy is shaped by top management and informs HR practices; HR practices are established by HR management, and both are implemented by line managers and influence climate. Individual differences might be subsumed under unit design because of their implications for selection and group composition. However, we differentiate variables under these headings according to the means by which managers influence them. Managers have control over unit design through composing, directing, and shaping work groups (i.e., task, work, and structure management). Individual differences by contrast are within the scope of managerial control through recruiting, selecting, and developing individual group members (i.e., personnel management) (Woods, Lievens, De Fruyt, & Wille, 2013). We have subsumed demographic variables under unit design because they are often associated with status differences (e.g., in organizations White and men are often accorded higher status), and status differences are shaped by the status hierarchy of an organization which is, at least to some extent, under the control of structure management (cf. Chattopadhyay, Tluchowska, & George, 2004). We did not include studies that examined identification or affective commitment (e.g., Randel & Jaussi, 2003; Van Der Vegt & Bunderson, 2005), salience (e.g., Randel, 2002), diversity experiences that take place outside of the organization (e.g., Lau & Murnighan, 2005), person‐group fit (e.g., Elfenbein & O'Reilly, 2007), and board of director diversity (Hafsi & Turgut, 2013) as a moderator of workplace diversity effects as these variables did not fall into any of the categories and, if at all, are only under the indirect control of managers (e.g., managers might facilitate identification via leadership).

## What Moderates the Effects of Workplace Diversity?

The Categorization‐Elaboration Model (CEM; van Knippenberg, De Dreu, & Homan, 2004) is the only comprehensive model of the contingencies of work group diversity effects, which is well supported by empirical evidence (e.g., Homan et al., 2008; Homan, Van Knippenberg, Van Kleef, & De Dreu, 2007; Kearney & Gebert, 2009; Kearney, Gebert, & Voelpel, 2009; see also van Dijk et al., 2012). Other approaches either received little empirical support, such as typological approaches (cf. van Dijk et al., 2012), or can be integrated with the CEM, such as the faultline approach (cf. van Knippenberg et al., 2004) and the contextual framework (cf. Joshi et al., 2011). While originally conceived as a model of work group diversity effects, there is nothing about the CEM that uniquely applies to work groups, and by implication, it may equally account for relational demography effects (cf. Guillaume, Dawson, et al., 2014) and organizational diversity effects (cf. Joshi et al., 2011). Thus, we review the workplace diversity literature through the lens of the CEM and examine to what extent the contingencies and processes stipulated by the model account for workplace diversity effects on performance, social integration, and employee well‐being variables.

The CEM defines diversity as “differences between individuals on any attribute that might lead to the perception that another person is different from self” (van Knippenberg et al., 2004, p. 1008). The CEM holds that diversity enfolds its effects via two routes that interact with each other: intergroup bias flowing from social categorization processes and information‐elaboration processes (van Knippenberg et al., 2004). Grounded in the social identity approach (Haslam, 2004; Hogg & Terry, 2000; Tajfel & Turner, 1986; Turner, Hogg, Oakes, Reicher, & Wetherell, 1987), the CEM defines social categorization in terms of people's tendencies to categorize similar others and self into in‐group and dissimilar others into out‐group; intergroup bias refers to more favorable responses toward in‐group than out‐group. Information‐elaboration is defined as the exchange, individual‐level process, discussion, and integration of information and perspectives. Diversity is expected to undermine social integration, well‐being, and performance through intergroup biases that are associated with negative affective‐evaluative reactions to dissimilar others and enhance performance through information‐elaboration.

The CEM suggests further that the extent to which diversity leads to positive or negative outcomes depends on three types of contingency factors: variables that render demographic differences salient, variables that engender or prevent intergroup bias, and variables that enhance or undermine information‐elaboration. Building on self‐categorization theory (Turner et al., 1987), social category salience is assumed to be a function of comparative fit (i.e., the extent to which the categorization results in between‐category dissimilarity and within‐category similarity), normative fit (i.e., the extent to which the categorization is meaningful), and cognitive accessibility (i.e., the ease to which the categorization comes to mind). In line with social identity theory (Tajfel & Turner, 1986), intergroup bias is proposed to be engendered by group members perceiving dissimilar others as a threat or challenge to a positive and distinct self‐image and undermine social integration, performance, and well‐being. Intergroup bias is suggested to disrupt information‐elaboration and group member ability and motivation to process rich and diverse information to facilitate it. Information‐elaboration is expected to enhance performance only in work groups performing tasks with a strong information‐processing or decision‐making component.

The social categorization processes and contingencies implied by the CEM are in line with relational demography theory, which argues that social categorization processes translate individual demographic dissimilarity into social integration outcomes and, in turn, well‐being and performance outcomes (Chattopadhyay, Tluchowska, et al., 2004; Tsui & Gutek, 1999). Research in relational demography does not speak to information‐elaboration processes but more recently has suggested that relational demography may under certain conditions motivate people to elaborate information more deeply (cf. Guillaume, Dawson, et al., 2014). Meanwhile, research has proposed that the CEM may also account for the effects of organizational diversity; organizational diversity may undermine organizational performance through lower relational coordination capabilities or social integration and enhance it through greater information‐processing and decision‐making capabilities (cf. Andrevski, Richard, Shaw, & Ferrier, 2014). Moreover, it has been suggested that the CEM can be taken to explain the effects of TMT diversity on organizational performance (cf. Joshi et al., 2011).

Thus, we argue that the contingencies associated with social categorization, intergroup bias, and information‐elaboration processes proposed by the CEM account for workplace diversity effects on social integration, well‐being, and performance‐related outcomes at the individual, group, and organizational levels. Variables that affect these contingencies are likely to moderate workplace diversity effects. In the following sections, we probe these ideas by examining how strategy, unit design, leadership, HR practices, climate and culture, and individual differences moderate the relationship between workplace diversity with social integration, performance, and employee well‐being‐related variables.

### Strategy

Research in workplace diversity examined the moderating effects of growth‐oriented, stability‐oriented, and customer‐oriented strategies and environments that are characterized by change, instability, uncertainty, complexity, and customer demographic diversity. While strategy variables seem particularly relevant to explain the effects of organizational diversity, it has been argued that they can also be viewed as an important contextual factor for work groups because strategy defines the amount of emphasis and resources devoted to organizational tasks (Jehn & Bezrukova, 2004). Even so, we found only one study that examined strategy as a moderator of work group diversity effects and none that looked at relational demography; most research is in organizational diversity. On the basis of the CEM, we would expect that organizations operating in growth‐oriented and customer‐oriented strategic environments are likely to benefit from diverse employee populations because they might enhance the capacity of organizations to innovate and adapt, and better understand customer needs (cf. Cox, 1993). Moreover, because growth‐oriented strategies are likely to promote openness towards new ideas and costumer‐oriented strategies tolerance towards a broader range of customers, they might promote the view that there is value‐in‐diversity (Jehn & Bezrukova, 2004; Richard, 2000) and facilitate social integration by eliminating intergroup bias and enhancing performance through more information‐elaboration. In a similar vein, workplace diversity might benefit organizations operating in environments characterized by change, instability, uncertainty, complexity, and high customer demographic diversity.

#### Work group diversity

Jehn and Bezrukova (2004) investigated how customer‐oriented, growth‐oriented, and stability‐oriented business strategies moderate the effects of work group diversity in terms of race, gender, functional background, age, level of education, and tenure on group performance and individual performance. No clear pattern of results emerged, and in most of the cases, strategy did not moderate the effects of work group diversity. One possible explanation for these findings might be that the range of the sample was too restricted; work groups that were sampled from the same organization and strategy is an organizational level variable that might vary very little within one organization.

#### Organizational diversity

Organizational strategy did moderate the effects of organizational diversity in the expected direction. Organizational diversity was positively related to organizational performance when organizations pursued a growth or innovation strategy but negatively when they had a low growth or low innovation strategy, or pursued a downsizing strategy (Dwyer, Richard, & Chadwick, 2003; Richard, 2000; Richard, McMillan, Dwyer, & Chadwick, 2003; Richard & Shelor, 2002). Similarly, Richard, Barnett, Dwyer, and Chadwick (2004) showed that entrepreneurial orientations that positively emphasized innovation and deemphasized risk‐taking, positively moderated nonlinear relationships between organizational diversity in terms of gender and racial diversity with organizational performance. Relatedly, Cunningham (2009) observed that the positive effects of racial diversity on organizational performance were strengthened in organizations that had a pro‐active diversity management strategy (i.e., diversity is valued, and diversity initiatives are incorporated in mission statement, policies, procedures, and practices).

The results for strategic environment variables were less clear. Supporting the idea that organizational diversity enhances organizational performance in growth‐oriented strategic environments, two studies showed that organizational diversity in terms of race was indeed positively related to organizational performance in munificent environments (supporting sustained growth) but negatively in resource‐scarce environments (undermining sustained growth; Andrevski et al., 2014; Richard, Murthi, & Ismail, 2007). In contrast, findings for the moderating role of conditions of environmental change on the temporal (tenure, age) and occupational (educational and occupational background) TMT diversity—performance relationship (Murray, 1989) and environmental instability on the racial diversity—productivity relationship (Richard et al., 2007), environmental uncertainty on the TMT demographic background—global strategic posture relationship (Cannella, Park, & Lee, 2008; Carpenter & Fredrickson, 2001) and strategic change on the TMT job‐related and non‐job‐related diversity—performance relationship (Naranjo‐Gil, Hartmann, & Maas, 2008), and the moderating effect of environmental complexity on TMT heterogeneity and Return on Assets (ROA) (Carpenter, 2002; Richard & Shelor, 2002) were inconclusive or not in the expected direction.

We found some support for the idea that organizational diversity might benefit organizations with a demographically diverse customer base. In one study, organizational racial/ethnic diversity was positively associated with sales performance in diverse but not in homogenous communities (Gonzalez, 2013). King et al. (2011) showed that the extent to which organizational demography in hospitals was representative of community demography had a positive effect on civility experienced by patients and organizational performance. Other studies found little support for the positive effects of employee–customer similarity in terms of race/ethnicity, age, and gender (Leonard, Levine, & Joshi, 2004) or the joint effects of racial diversity and community demographics on performance (Sacco & Schmitt, 2005).

#### Summary

Taken together, these findings suggest that growth‐oriented and diversity management strategies positively moderate the effects of organizational diversity on performance. This is likely because these strategies might promote the view that there is value‐in‐diversity facilitating the elaboration of task relevant information and in turn leading to innovation and better decision‐making. A downsizing strategy may undermine performance because it might engender threat rendering demographic differences salient and eliciting intergroup bias, which in turn might lead to lower social integration. That environmental variables including change, instability, uncertainty, and complexity produced mixed results may be accounted for by higher‐order contingencies. We would expect that higher information‐processing or better decision‐making capabilities associated with greater workplace diversity should benefit organizations in such environments, but it may require a growth‐oriented or diversity management strategy to unlock the positive effects of workplace diversity. The inconsistent findings for customer demographic diversity may also be accounted for by a higher‐order interaction effect between workplace diversity, customer demographic diversity, and customer‐oriented strategy: only when there is a customer‐oriented strategy that encourages employees to see value‐in‐diversity will workplace diversity lead to a better understanding of a diverse customer base and in turn enhance performance. Little is known about what strategy variables might moderate the effects of work group diversity and relational demography. With the only study speaking to the issue (Jehn & Bezrukova, 2004) suffering from range restriction, it may well be that future studies that sample work groups from a wide range of organizations will find support for the idea that strategy moderates work group diversity and relational demography effects.

### Unit design

Research examined a variety of unit characteristics as moderators of workplace diversity effects, which fell into three broad categories: compositional variables other than workplace diversity (e.g., demographics, type of diversity, faultline and subgroup strength, and size), task design (e.g., task characteristics, such as task complexity and task novelty; type of team; interdependence; collocation; autonomy; empowerment; decentralization; meeting informality; and decision support systems), and lifecycle/temporal variables (e.g., group member tenure, team longevity, and time). Faultline strength (extent to which multiple demographic attributes align to form homogenous subgroups) and status differences that are often associated with membership in different demographic subgroups in organizations (e.g., women and non‐white are often accorded lower status than men and White) have been linked to greater social category salience and intergroup bias and should thus lead to negative outcomes (van Knippenberg et al., 2004; see also Chattopadhyay, Tluchowska, et al., 2004). Similarly, size has been suggested to enhance social category salience (Wegge et al., 2008). On the basis of the CEM, it can be expected that diversity benefits in particular organizations and work groups that are concerned with innovation and complex tasks. Building on the contact hypothesis (Pettigrew, 1998), research in diversity has been arguing that interdependence (extent to which goal, reward, and task structures promote cooperation), collocation, and decision support systems may promote positive intergroup contact and invite information exchange and discussion and should therefore lead to positive outcomes (cf. van Knippenberg & Schippers, 2007). One may expect that autonomy, empowerment, decentralization, and possibly meeting informality (degree to which meetings are planned and structured) moderate workplace diversity effects positively because these factors are generally seen to facilitate participation and inclusion and might therefore eliminate intergroup bias and facilitate information‐elaboration (cf. Avery, Wang, Volpone, & Zhou, 2013). Temporal and lifecycle variables, such as group member tenure, team longevity, and time, might positively moderate workplace diversity effects because it likely takes time to overcome stereotype‐based impressions and uncover unique information, knowledge, and perspectives associated with workplace diversity (van Knippenberg & Schippers, 2007).

#### Relational demography

Research in relational demography provides support for the idea that the effects of relational demography on social integration and performance outcomes become less negative over time (Chatman & Flynn, 2001; see also Sacco & Schmitt, 2005) and when interdependence is high instead of low (for a meta‐analysis, see Guillaume et al., 2012). Even so, the meta‐analysis also reports a significant amount of variance unaccounted for by the moderator. One explanation for these findings might be that simple demographic attributes are often associated with subgroup status differences in organizations and might moderate the effects of demographic dissimilarity on work outcomes because identification with a work group dominated by high status but not low status group members allows group members to derive a positive social identity and enhance their self‐esteem (Chattopadhyay, Tluchowska et al., 2004). This is generally corroborated by the evidence as far as social integration and well‐being outcomes are concerned and as long as people's actual belief systems about the permeability, stability, and legitimacy of the status hierarchy are taken into account as a moderator (Chattopadhyay, 2003) or when interpreting the results (e.g., Chatman & O'Reilly, 2004; Chattopadhyay, 1999; Chattopadhyay, Finn, & Ashkanasy, 2010; Chattopadhyay, George, & Shulman, 2008; Choi, 2013; Paletz, Peng, Maslach, & Erez, 2004; Tsui et al., 1992).

For performance outcomes, the effects are inconclusive (e.g., Joshi, Liao, & Jackson, 2006; Kirchmeyer, 1995; Paletz et al., 2004) even when three‐way interactions between relational demography, demographic status, and work group or unit level diversity are taken into account (e.g., Brodbeck, Guillaume, & Lee, 2011; Elvira & Cohen, 2001; Joshi et al., 2006). Meanwhile, Avery et al. (2013) examined the moderating effect of gender dissimilarity on the empowerment–performance relationship and found, contrary to what one would expect, that relational demography is negatively related to individual empowerment and in turn individual performance when team empowerment is high instead of low. Relatedly, the effects of functional dissimilarity on involvement in decision‐making were positive when power centralization was high and negative when it was low (Bunderson, 2003). A complicating factor in the relationship between relational demography and (performance) outcomes might be that interacting with demographically dissimilar others may not only inspire concerns for a positive social identity (cf. self‐enhancement perspective; Chattopadhyay, Tluchowska, et al., 2004) but also increase uncertainty about how to interact with dissimilar others and motivate people to reduce the uncertainty (cf. uncertainty reduction perspective; Chattopadhyay, George, & Lawrence, 2004; Chattopadhyay, George, & Ng, 2011). Thus, high autonomy and low power centralization might have resulted in more uncertainty about how to interact with demographically dissimilar peers.

More recently, relational demography theory has therefore begun to integrate both perspectives with theories on self‐regulation to clarify the conditions under which concerns for self‐enhancement and uncertainty reduction might become more prevalent (Chattopadhyay, George, & Ng, 2015). Guillaume, van Knippenberg, and Brodbeck (2014) argued that contingent on status (cf. concerns for self‐enhancement) demographic dissimilarity might promote self‐regulatory behaviors aimed at reducing the uncertainty, and while this might enhance performance at lower and moderate levels, they suggested it decreases performance at higher levels (i.e., a curvilinear relationship) due to the increasing risk of self‐regulatory failure. Supporting this notion, the authors found that cultural dissimilarity had a decreasingly positive effect on individual performance for low‐status group members and an increasingly negative effect for high‐status members, which was mediated by performance monitoring, a form of social self‐regulation aimed at reducing uncertainty by meeting performance standards and peer expectations.

#### Work group diversity

Task characteristics were found to be important moderators of the effects of work group diversity on work group performance. In the most up‐to‐date meta‐analysis on the performance effects of work group diversity, van Dijk et al. (2012) showed that demographic work group diversity was associated with more work group performance when the outcome was innovation or when the diversity characteristic was associated with task‐relevant knowledge (which seems to be more likely the case for functional background, education, and tenure than other demographic diversity characteristics). Meanwhile, Díaz‐García, González‐Moreno, and Sáez‐Martínez (2013) observed that although work group gender diversity was positively related to radical innovation, it did not promote incremental innovation in the same way, further corroborating the idea that the higher the degree of novelty of the task the more likely will work groups benefit from demographic diversity. Relatedly, findings by Wegge et al. (2008) support the idea that work group diversity may enhance performance (and health) particularly on complex tasks. The meta‐analysis by van Dijk et al. (2012) found further that team size, type of diversity (i.e., separation, variety and disparity), study setting, industry setting, and team type did not moderate the effects of diversity on work group performance.

Workgroups can be composed so that multiple demographic attributes align to form faultlines (e.g., two female nurses/two male doctors) or cross‐cut so that attributes are uncorrelated (e.g., male/female nurses, male/female doctors). While both types of diversity render demographic differences salient, faultlines but not cross‐cutting categories engender intergroup bias: a recent meta‐analysis found that demographic faultline strength is negatively related to social integration and, in turn, performance (Thatcher & Patel, 2011). Even so, other researches corroborate the notion that faultlines engender social categorization and render diversity more salient, but the effects on intergroup bias are moderated by situational variables that promote positive or negative views towards diversity (e.g., Homan, Greer, Jehn, & Koning, 2010; Homan et al., 2008). Similarly, research showed that strong faultlines stemming from large status differences between demographic subgroups in work groups undermine social cohesion and work group performance when intergroup relations between the subgroups in the wider social context are negative but enhance cohesion and performance when intergroup relations are positive (Leslie, 2014).

Earlier findings with regard to the moderating role of interdependence and time/team tenure have been inconclusive (van Knippenberg & Schippers, 2007). One explanation accounting for these inconsistent findings may be the reformulated contact hypothesis (Pettigrew, 1998), which can be taken to suggest that time/team tenure and interdependence interact with each other and lead to positive intergroup contact and thus more social integration and higher performance only in situations marked by equal status and “authorities, law, or customs” promoting positive views towards diversity and that it takes time for these positive effects to materialize. Indeed, Joshi and Roh's (2009) meta‐analytic findings point toward a three‐way interaction between work group diversity, interdependence/team tenure, and unequal demographic subgroup status on group performance. Alternatively, interdependence might be beneficial only at earlier stages of group formation to overcome low levels of social integration. Mohammed and Angell (2004) found that team orientation (cf. interdependence) negatively moderated the effects of gender (but not ethnic) diversity on relationship conflict at time 1 but not any longer at time 2. Schippers, Den Hartog, Koopman, and Wienk (2003) found a three‐way interaction between work group diversity, group longevity, and outcome interdependence on satisfaction and commitment but not on performance.

Two studies examined the moderating role of team autonomy. Contrary to expectations, autonomy seems to be detrimental to the functioning of diverse teams. Rico, Molleman, Sánchez‐Manzanares, and Van der Vegt (2007) found that strong‐faultline teams performed worse and reported lower levels of social integration than did weak‐faultline teams under high but not low team task autonomy conditions. Similarly, Molleman (2005) reported that team autonomy negatively moderated the effects of ability faultline strength on team cohesion and personality faultline strength on intra‐team conflict but not the effects of demographic faultline strength. As with relational demography, these findings seem to point towards uncertainty as yet another mechanism by which work group diversity enfolds its effects. High levels of autonomy might increase uncertainty on how to interact with demographically dissimilar peers. Research showing that culturally diverse work groups outperform homogenous groups when they utilized a group decision support system (Daily, Whatley, Ash, & Steiner, 1996) might also be interpreted through the lens of an uncertainty reduction perspective. Group decision support systems facilitate group interaction and decision‐making through individual idea generation and structured group idea evaluation, which might not only decrease intergroup bias and facilitate information‐elaboration but also reduce the uncertainty by structuring interpersonal conduct.

#### Organizational diversity

At the organizational level, temporal variables produced mixed results: Choi and Rainey (2010) reported that gender diversity had a positive effect on performance when employees worked longer together but a negative effect for racial diversity and no effect for age diversity. Barkema and Shvyrkov (2007) found the relationship between TMT diversity (in terms of tenure/strong faultlines but not for education) and strategic innovation to be negatively moderated by overlapping team tenure (positive/negative at first then no effects). Boerner, Linkohr, and Kiefer (2011) showed that longevity moderated the effects of age, tenure, and educational and industry diversity on some but not all performance outcomes and in an inconclusive pattern. Richard, Ford, and Ismail (2006) observed a positive moderation of the organizational life cycle on the effect of racial and gender diversities on organizational performance (positive first then negative). Higher‐order interaction effects might therefore be operating here too. As a case in point, Carpenter (2002) showed that team tenure moderated the effects of TMT diversity on firm performance negatively only when these firms were operating in hostile business environments. Further, we found support for task complexity and collocation moderating the effects of diversity on performance positively. Ali, Kulik, and Metz (2011) showed that organizational gender diversity produced (decreasingly) positive effects on employee productivity in the service industry (where more complex tasks requirements might benefit from diversity) but no effect in manufacturing (where less complex task requirements might benefit less from diversity), and Cannella et al. (2008) reported that the TMT functional diversity‐firm performance (ROA) relation became more positive as the proportion of TMT members with offices in the same location increased.

Decentralization positively moderated the relationship between TMT diversity and firm performance (Boone & Hendriks, 2009; Richard & Shelor, 2002). Given higher decentralization implies less TMT autonomy because responsibility for decision‐making is delegated downward and across organizational levels; this seems to echo our earlier findings for relational demography and work group diversity that autonomy and empowerment taken on their own may spawn negative diversity effects due to higher levels of uncertainty. In line with these ideas, van Knippenberg, Dawson, West, and Homan (2011) report that clear and shared objectives attenuated the negative effects of some TMT faultlines (gender*function, gender*tenure but not function*tenure, and gender*function*tenure) on firm productivity (but was unrelated to profitability), which may be taken to suggest that clear and shared goals are an effective mean to reduce the uncertainty. Meeting informality (degree to which meetings are planned and structured) produced mixed results, however (Tuggle, Schnatterly, & Johnson, 2010). Informality negatively moderated the effects of functional diversity on discussion of entrepreneurial issues but positively for output‐oriented and industry background diversity and strong faultlines; no effects were found for tenure diversity and weak faultlines. Even so, when combined with high levels of involvement of racio‐ethnic minorities, employee empowerment systems positively moderated the racio‐ethnic diversity—innovation relationship (Yang & Konrad, 2011). It might well be that such conditions foster the development of an organic social identity that promotes positive intergroup relations and clarifies interpersonal conduct satisfying people's need for self‐enhancement and uncertainty reduction (Haslam, Eggins, & Reynolds, 2003; Hornsey & Hogg, 2000a, 2000b).

#### Summary

We have found that faultlines, cross‐categorization, and status differences between demographic subgroups render diversity salient. Cross‐categorization was shown to prevent intergroup bias and facilitate social integration, performance, and well‐being. Whether faultlines and subgroup status differences lead to intergroup bias and undermine social integration, performance and well‐being seem to depend on whether situational variables promote negative or positive intergroup relations. Team size and type of diversity (i.e., separation, variety, and disparity) did not moderate the effects. Further, our findings show that for work group diversity task characteristics matter. Any type of demographic diversity in work groups can facilitate innovation but only when demographic diversity is associated with task‐relevant knowledge does it enhance performance of teams performing complex tasks. Team type and industry setting do not seem to play a role. Little is still known as to whether these findings generalize to relational demography and organizational diversity effects. Supporting this notion, there is some evidence that relational demography might under certain conditions enhance creativity (e.g., Chatman et al., 1998; Choi, 2007) and, as we saw earlier, that demographic diversity may benefit organizations operating in growth‐oriented strategic environments.

Considering higher‐order contingency factors and nonlinear relationships seems also relevant: positive effects of diversity are likely to emerge only on knowledge‐based and innovation tasks and when people have the ability and motivation to accomplish them; it may take demographically dissimilar people more time to coordinate their interactions and make effective use of their different Knowledge, Skills, and Abilities (KSA); and, when demographic differences are vast, they may exceed individuals' capacity to self‐regulate their behavior. We found some support for the idea put forward by the reformulated contact hypothesis (Pettigrew, 1998) that positive contact in work groups occurs only in situations marked by cooperative interdependence, equal subgroup status, and/or “authorities, law, or customs” promoting positive views towards diversity but that it takes time for the effects to materialize. A complicating factor here might be that subgroup status differences might not always have negative effects. Working in a group dominated by a high‐status subgroup might motivate group effort of low‐status subgroup members (cf. Chattopadhyay, Tluchowska, et al., 2004), and status differences when perceived as veridical, stable, and legitimate might enhance social integration and performance (van Dijk & van Engen, 2013).

An interesting finding is that situations characterized by autonomy may aggravate rather than alleviate negative workplace diversity effects; decision support systems, shared objectives, and clear roles suppress negative and facilitate positive workplace diversity effects. On a conceptual level, these findings imply that the CEM may have to accommodate more explicitly for the notion that diversity and dissimilarity not only raise concerns about maintaining a positive group identity but also lead to greater uncertainty about how to interact with demographically dissimilar peers (cf. Chattopadhyay et al., 2011). This may be taken to suggest that effective workplace diversity management does not only depend on structures that facilitate information‐elaboration and positive intergroup contact but also reduce uncertainty. Next to clarifying roles (Dovidio, Gaertner, & Validzic, 1998), building a shared superordinate identity that allows people independent of their demographic background to derive a positive and distinct identity (Haslam et al., 2003; Hornsey & Hogg, 2000a, 2000b), and the use of decision support systems might be an effective means to reduce both intergroup bias and uncertainty and facilitate information‐elaboration.

### Human resource management practices

Relatively few studies have used HR practices as moderators of workplace diversity effects on social integration, well‐being, and performance‐related variables: in fact, just five papers were found in our search, four of which focused on the work group level and one on the organizational level. This is surprising as HR practices are seen as one of the keys to the effective management of workplace diversity, with the suggestion that the diverse groups and workforces are more likely to have positive outcomes when people management practices are designed to promote their benefits (cf. Avery & McKay, 2010).

#### Work group diversity

The only study that found a moderating effect sampled work groups from 35 organizations showing that the inverted curvilinear effects of higher tenure diversity on team innovation were attenuated with more team‐oriented HR practices (i.e., teamwork training, team‐based rewards, teamwork, feedback systems, and participation programs; Chi, Lin, & Huang, 2009). Ely (2004) sampling work groups from a single organization report that the level of participation in diversity education programs reduced the negative effects of gender diversity on one performance outcome (number of customer referrals) but not on other performance outcomes (sales revenue, customer satisfaction, sales productivity, and total performance) or the negative effects of race and gender diversity on these outcomes. Similarly, Jehn and Bezrukova (2004) sampled work groups from a single organization and found little support that HR practices (i.e., training‐oriented and diversity‐oriented HR practices) moderate the relationship between age, gender, race, tenure, functional, and educational diversity with individual and group performances and bonuses. Training‐oriented but not diversity‐oriented HR strategy attenuated the negative effects of education diversity on group performance and both training‐oriented and diversity‐oriented HR practices attenuated the negative effects of gender and educational diversity on bonuses. HR practices did not moderate any of the other relationships. Homan, Buengeler, Eckhoff, Van Ginkel, and Voelpel (2015) examined the interactive effects of diversity training, diversity beliefs and nationality diversity on team creativity in student teams. Based on the CEM, they instructed team members how to harness nationality diversity for team creativity. Results showed that diversity training (compared with control training) enhanced team creativity when team members held less positive diversity beliefs, and nationality diversity was high but undermined it when nationality diversity was low and had less impact when team members held positive diversity beliefs. Team efficacy mediated the effects.

#### Organizational diversity

The one paper that examined organizational level effects was a study by Choi and Rainey (2010) of the effects of demographic diversity on organizational performance, with diversity management practices as a moderator. The effects were inconclusive; perceptions of diversity management moderated the effects of racial diversity on organizational performance positively but did not moderate the effects of age and gender diversity. For objective diversity management (number of Equal Employment Opportunity (EEO) complaints), gender diversity had a positive effect on performance when diversity was not managed effectively, while racial and age diversity were related positively to performance when diversity was managed effectively.

#### Summary

We found evidence that diversity training for teams building on the principles of the CEM enhances performance on a creativity task. Overall, however, these papers give little clear evidence for the nature of the moderating effects of HR practices on work group and organizational diversity effects on social integration, well‐being, and performance. Nothing is known about the moderating effects of HR practices on relational demography effects. Clearly, this is an area where more research is needed. Next to identifying a broader range of specific HR practices (staffing, appraisal, rewards, and promotions; cf. Avery & McKay, 2010) that might moderate workplace diversity effects, we also need to better understand how these practices interact with each other. Research in high‐performance HR management practices (Iverson, Zatzick, & McCrae, 2008) makes it clear that only synergistic and mutually reinforcing bundles of HR practices that are well aligned with an organization's strategy enhance performance, innovation, and well‐being.

On the basis of the CEM and more recent work on diversity mindsets (van Knippenberg, Van Ginkel, & Homan, 2013), we would expect that HR practices that build relational coordination capabilities are likely to promote social integration and well‐being. HR practices that build information‐processing and decision‐making capabilities may enhance innovation and decision‐making quality. Because such capabilities are best developed in the context in which they are applied, collective or team‐based trainings might be more effective than individual trainings. A complicating factor here might be that the extent to which HR practices are effective might depend on whether employees accept them. Future research might therefore want to consider employees' attitudes (e.g., identification with HR practices) toward HR practices as a higher‐order contingency factor. Further, diversity‐related HR practices, such as diversity training, might lead to negative effects when workplace diversity is low as people might come to realize that they do not possess the necessary resources to succeed.

### Leadership

Although leadership has been noted to be an important contextual variable to aid our understanding of the workplace diversity to outcome relationship (Joshi et al., 2011; van Knippenberg et al., 2004), a limited number of studies exist that have examined leadership as a moderator. Most of these studies examined work group diversity effects; very few looked at relational demography and organizational diversity effects. On the basis of the CEM, it would be expected that leadership will positively moderate workplace diversity effects on social integration, well‐being, and performance‐related variables when it fosters identification with the work group or organization and facilitates the elaboration and integration of differences in expertise and perspectives (Kearney & Gebert, 2009).

#### Relational demography

Research in relational demography examined supervisor facilitation, leader openness, and leader–follower similarity. Pelled, Xin, and Weiss (2001) reported that high levels of supervisor facilitation attenuated a negative relationship between tenure dissimilarity and conflict (task and emotion) but did not so for age dissimilarity. Troester and van Knippenberg (2012) observed that leader openness and leader–member similarity (nationality) were more positively related to leader‐directed voice when relational demography was high rather than low (both mediated by psychological safety and affective commitment). This can be taken to suggest that relational demography effects are likely more positive when leaders are more similar to team members or high on openness.

#### Work group diversity

Most studies in work group diversity looked at transformational leadership finding in the majority of cases that diverse teams are better off with transformational leaders: Diversity was more positively or less negatively related to collective team identification and team performance (Kearney & Gebert, 2009), teams' productive energy (Kunze & Bruch, 2010), and team creativity (Shin & Zhou, 2007). Even so, in one study transformational leadership did not reduce the negative effect of gender diversity on team commitment (Seong & Hong, 2013). Accounting for this inconsistent finding might be higher‐order contingencies. Greer, De Hoogh, Den Hartog, and Homan (2012) found that the interactive effects of work group diversity were not only contingent on visionary leadership but also leaders' tendency to categorize team members into in‐group and out‐group: when leaders exhibited high levels of visionary behavior and did categorize team members into in‐groups and out‐groups, ethnic team diversity was related negatively to team communication and team financial performance but positively when the leader did not.

Alternatively, heterogeneous and homogenous teams might benefit from different leadership styles, which might further depend on task requirements. Homan and Greer (2013) showed that diverse teams prefer a considerate leadership style and function more effectively when leader consideration is high instead of low. Somech (2006) reported that a participative but not a directive leadership style is positively associated with team innovation (through more team reflection) in high but not low functionally diverse teams; however, participative leadership decreased team in‐role performance in high functionally diverse teams. Nishii and Mayer (2009) proposed that through the pattern of Leader‐Member‐Exchange (LMX) relationships that team leaders develop with followers, they influence inclusion and status differentials within groups such that high LMX with all group members safeguard against negative diversity effects. This notion was partially corroborated by their findings: Higher LMX (mean) decreased the positive effects of tenure and demographic diversity on turnover. Higher LMX differentiation exacerbated the positive effects of tenure and demographic diversity on turnover. A three‐way interaction between LMX (mean), LMX differentiation, and demographic (but not tenure) diversity on turnover was also found such that the interaction between demographic diversity and LMX differentiation was only significant when LMX mean was high. For work group performance, Stewart and Johnson (2009) found a different pattern of results: LMX differentiation was positively associated with work group performance when aggregate LMX was high (above the median); among less gender diverse groups, LMX differentiation was not associated with performance when aggregate LMX was high. No effects were found for functional diversity. One study looked at the joint effects of leader demographics and work group diversity on performance; leader gender but not leader ethnicity and tenure moderated the effects (Jackson & Joshi, 2004).

#### Organizational diversity

Shared experiences between leaders and team members lead to positive effects of TMT functional diversity on firm performance (Buyl, Boone, Hendriks, & Matthyssens, 2011) and attenuated negative effects of TMT tenure diversity on combat performance (Soojin, Keunyoung, Seokho, & Sungzoon, 2013). Choi (2013) observed opposite to what had been expected that supervisors' support weakened the positive relationship between managerial diversity and job satisfaction, suggesting that in organizations that maintain greater support from supervisors, managerial diversity is less strongly positively related to job satisfaction of employees.

#### Summary

Research corroborates the idea that transformational leadership (and related behaviors such as leadership consideration and participative leadership) safeguards against negative work group diversity effects on social integration variables and facilitates performance on knowledge and innovation tasks. Even so, as leaders may have a tendency to categorize group members into in‐groups and out‐groups and develop transformational relationships only with in‐group members, future research might want to consider next to transformational leadership (or related leadership behaviors) other variables that facilitate inclusive leadership behavior (e.g., leader openness, leader diversity beliefs, and leader empathy). Such variables might also account for the inconsistent effects of LMX differentiation; differentiated LMX relationships in diverse work groups might facilitate social integration, performance, and well‐being as long as they are perceived to be based on fair and equal treatment of all group members. Little is still known whether these findings generalize to relational demography and organizational diversity.

In light of recent calls to develop more clearly defined and empirical distinct leadership concepts (van Knippenberg & Sitkin, 2013), one may argue on the basis of the CEM that effective leadership of diverse work groups needs to serve two functions: building an inclusive superordinate identity that facilitates positive intergroup contact and promoting a thorough consideration of all available task‐relevant resources to encourage information‐elaboration. Accordingly, leadership that promotes positive intergroup contact, advocates for diversity as an informational resource, stimulates information‐elaboration, and engenders team reflexivity might turn out to be an effective means to manage workplace diversity (van Knippenberg et al., 2013). A complicating factor here might be leader–follower demographic dissimilarity as it may render leaders, in particular those with a low‐status demographic background, less prototypical. More research into how dissimilar leaders can establish their group prototypicality thus seems also important (cf. Rast, Gaffney, Hogg, & Crisp, 2012).

### Climate and culture

Research in workplace diversity suggests that through their persuasive effects on individual, group, and organizational behaviors, climate and culture variables can both, safeguard against the negative effects of workplace diversity on social integration outcomes and performance by eliminating intergroup bias and enhance performance in demographically diverse work groups working on innovation and complex tasks by facilitating information‐elaboration (cf. Avery & McKay, 2010; van Knippenberg & Schippers, 2007). The studies we found examined a wide range of culture and climate variables as moderators; most of these studies looked at work group diversity.

#### Relational demography

Relational demography effects on productivity and creativity (Chatman et al., 1998) as well as individual cooperative behaviors (Chatman & Spataro, 2005) were found to be more positive in work groups that had a collectivistic instead of an individualistic culture. In a related way, supportive (diversity) climate was found to negatively moderate the effects of gender dissimilarity (but not racial/ethnic dissimilarity) on intention to quit but have no effect on organizational commitment or identification (Gonzalez & DeNisi, 2009). They also report a three‐way interaction between racial/ethnic dissimilarity, race/ethnicity, and diversity climate on intention to quit with Hispanics more likely to leave when they are dissimilar and when the diversity climate is favorable. However, in another study, supportive climate did not moderate an inverse curvilinear relationship between racial diversity and social support (Bacharach, Bamberger, & Vashdi, 2005).

#### Work group diversity

Trust, psychological safety, and related variables were found to promote positive interpersonal relations and prevent negative performance effects in demographically diverse work groups. Trust attenuated the negative effects of functional diversity on performance (when rated by managers but not by the team) (Peters & Karren, 2009). Psychological safety enhanced the performance in diverse organizational communities of practice (Kirkman, Cordery, Mathieu, Rosen, & Kukenberger, 2013) and attenuated negative diversity effects (both in geography and nationality) in aerospace design teams (Gibson & Gibbs, 2006). Perceived interpersonal injustice (but not other forms of injustice) moderated the effect of faultline strength on anxiety and depression (Bezrukova, Spell, & Perry, 2010); when people perceived low instead of high injustice, the positive effects of weak faultlines on anxiety and depression were reduced. Fay, Borrill, Amir, Haward, and West (2006) observed that better team processes (measured with the Team Climate Inventory; Anderson & West, 1998) in terms of vision, participation safety, task orientation, and interaction frequency in teams meant that there was a positive effect of professional diversity on innovation, but where team processes were poor, there was no link.

Mindsets that facilitate information‐elaboration have also been found to be important. Kooij‐de Bode, van Knippenberg, and van Ginkel (2008) report that ethnically diverse groups benefit from instructions emphasizing information integration in particular when dealing with distributed information. Gilson, Lim, Luciano, and Choi (2013) observed that tenure diversity positively influenced individual knowledge (and subsequently creativity) at high levels of knowledge sharing but negatively at low levels. Similarly, perspective taking was found to positively moderate the relationship between diversity of perspectives and team creativity (Hoever, van Knippenberg, van Ginkel, & Barkema, 2012). Richter, van Knippenberg, Hirst, and Baer (2012) examined the effects of functional diversity on individual creativity in Research and Development (R&D) teams; they found an important role of “knowledge of who knows what”. As knowledge of who knows what increases, the effect of diversity on creativity increases as long as creative self‐efficacy is high (a three‐way interaction).

One study spoke to the idea that work group diversity might also evoke uncertainty and that clear norms of interpersonal conduct might reduce it. Goncalo, Chatman, Duguid, and Kennedy (2014) showed in two group experiments that the norm to be politically correct promotes rather than suppresses team member creativity by reducing the uncertainty they experience in mixed‐sex work groups. This corroborates our earlier findings that uncertainty reduction might be yet another process through which diversity unfolds its effects. In contrast, the findings for the moderating role of team coordination was inconclusive; it moderated the diversity–performance link for different types of diversity (national, functional, and gender) but not in a consistent direction (the effect of functional diversity becoming more positive as coordination increases but the others becoming more negative; Zoogah, Vora, Richard, & Peng, 2011).

Surprisingly, only two studies examined the moderating role of diversity climate and climate for inclusion. Nishii (2013) reported that an inclusive climate reduces the negative effect of gender (but not age, tenure, and education) diversity on team conflict and, in turn, the negative effect of team member satisfaction on turnover. Drach‐Zahavy and Trogan (2013) found that team diversity was related to less interpersonal aggression in units with a high diversity climate for tenure and ethnic diversity but not for sex and age diversity. Not much progress has been made with regard to cooperative norms and values, which have previously been reported to produce mixed results (cf. van Knippenberg & Schippers, 2007). A more recent study showed that cooperative norms attenuated the negative effect of gender diversity on team commitment (Seong & Hong, 2013).

#### Organizational diversity

Research in organizational diversity looked at support, justice, positive debate, and cooperation climate variables. Choi (2013) found that racial/ethnic diversity in managerial teams was negatively related to job satisfaction but that this effect was lessened in agencies that maintain higher levels of justice and support for subordinates but enhanced in agencies with a positive diversity climate. Similarly, Gonzalez and DeNisi (2009) found that both racial/ethnic and gender diversity had a negative association with productivity and Return On Investment (ROI) under adverse diversity climate conditions, but the effect was positive under favorable conditions. Diversity had an inverse U‐shaped relationship with productivity and return on profit when diversity climate was supportive but U‐shaped when not. Positive debate processes in functionally and educationally diverse teams enhanced profit but not sales (and there was no effect with tenure diversity; Simons, 1995), while collaborative behavior alongside information exchange in functionally diverse TMTs was found to enhance firm sales performance (Boone & Hendriks, 2009). Results for the moderating role of cooperation and results‐oriented cultures were inconclusive (Choi & Rainey, 2010): cooperation positively moderated the effects of racial but not gender or age diversity on performance in US federal agencies, and high results‐oriented cultures positively moderated the effects of age but not racial or gender diversity on performance.

#### Summary

There are two types of moderators that appear to have positive impacts on the effects of diversity across multiple studies: (shared) perceptions of psychological safety/trust/justice promoting social integration and well‐being via positive intergroup contact and (shared) perceptions that encourage information sharing and integration enhancing performance on complex tasks through information‐elaboration. We found little support for the moderating role of diversity climate, however. This is in line with recent calls to move beyond diversity climate and focus on diversity mindsets instead (van Knippenberg et al., 2013). Diversity mindsets clarify diversity‐related goals and procedures how to achieve these goals with the two main goals being the elimination of intergroup bias and, if the task demands, facilitation of information‐elaboration. These mindsets are thought to positively moderate the effects of diversity when they are accurate, shared, and when there is awareness of sharedness. As such, diversity mindsets might also be an effective means to reduce social uncertainty in diverse work groups by clarifying interpersonal conduct.

Results for cooperative values and norms, such as cooperation/collectivism, were mixed in particular for studies on work group and organizational diversity. One explanation for these inconsistent findings could be that simply promoting cooperative norms and values may at times suppress apparent differences between demographic subgroups and aid little in resolving conflicted intergroup relations let alone promote information‐elaboration. In contrast, political correctness norms that clarify interpersonal conduct between demographic subgroups were found to positively moderate the effects of diversity on performance on a creativity task. As per our earlier arguments, an effective diversity culture is therefore likely one that allows all employees independent of their demographic background to derive a positive and distinct social identity and clarifies norms of interpersonal conduct (Haslam et al., 2003; Hornsey & Hogg, 2000a, 2000b).

### Individual differences

Differences in individual psychological factors are obvious targets for research into moderation of the relations of diversity and organizational outcomes. Indeed, the CEM (van Knippenberg et al., 2004) suggests that individual differences moderate the effects of workplace diversity on intergroup bias (e.g., stereotypes, diversity beliefs, attitudes, and values) as well as information‐elaboration (e.g., intelligence, task‐specific knowledge, skills, and abilities, communication skills, and individual differences in information‐processing motivation). Thus, we would expect that the effects of workplace diversity on social integration, well‐being and performance‐related variables are contingent on these individual differences. We could identify no studies examining individual difference moderators of organizational diversity effects, and so we restrict our review to relational demography and work group diversity.

#### Relational demography

Personality variables moderated relational demography effects on social integration and performance outcomes. Flynn, Chatman, and Spataro (2001) reported that demographically dissimilar people high on Extraversion and self‐monitoring traits were perceived less negatively; favorable impression formation, in turn, was positively related to social integration and individual performance. Stereotypes also moderated the effects of relational demography on health outcomes. Liebermann, Wegge, Jungmann, and Schmidt (2013; see also Avery, McKay, & Wilson, 2007) reported that the relationship between age dissimilarity and health was negative for older and younger workers who held less favorable age stereotypes towards age dissimilar others but unrelated for those holding more favorable age stereotypes. We also found that the need for uncertainty reduction of group members moderated the effects of race dissimilarity on group cohesiveness but not for gender dissimilarity and other outcomes, such as identification and liking, (Goldberg, Riordan, & Schaffer, 2010) lending some support to the idea that relational demography might not only evoke concerns for a positive and distinct social identity but also for uncertainty reduction.

#### Work group diversity

In studies on individual differences as moderators of work group diversity, the effects of aggregated individual differences are usually examined with respect to team‐level outcomes. We found that Openness positively moderated the effects of salient faultlines on information‐elaboration and team performance in diverse teams performing a decision‐making task (Homan et al., 2008). Greater need for cognition (tendency to engage in cognitive activity) enhanced collective team identification, information‐elaboration, and team performance in diverse teams performing knowledge‐based tasks (Kearney et al., 2009). Pro‐diversity beliefs enhanced information‐elaboration and work group performance in diverse teams that worked on a task with a strong information processing component (Homan, Van Knippenberg, et al., 2007). Similarly, other studies showed that work group diversity had more positive effects on social integration outcomes (van Dick, van Knippenberg, Hägele, Guillaume, & Brodbeck, 2008; van Knippenberg, Haslam, & Platow, 2007), information‐elaboration (van Dick et al., 2008), and creativity (Nakui, Paulus, & Van Der Zee, 2011) when group members hold pro‐diversity beliefs. Diversity beliefs also lead work group members to construe diversity in terms of individual differences rather than subgroups (Homan, Van Kleef, De Dreu, & van Knippenberg, 2007).

Nederveen Pieterse, van Knippenberg, and van Dierendonck (2013) found in two studies that cultural diversity is more positive for information‐elaboration and, in turn, team performance with higher learning approach orientation (motivation to learn and improve) and lower performance avoidance orientation (motivation to avoid failure and negative evaluation). Relatedly, Meyer and Schermuly (2012) found a three‐way interaction between diversity beliefs, task motivation, and faultlines (in terms of gender, age, and educational background). Only when group members hold pro‐diversity beliefs and had high task motivation were faultlines positively related to team performance. Further, creative self‐efficacy facilitated creativity in diverse teams with greater informational resources and when groups member had knowledge of who knows what (Richter et al., 2012), and group efficacy positively moderated the effects of gender diversity on group performance but not on social integration (Lee & Farh, 2004). Moreover, we found one study that examined the moderating effect of social competence. Meyer, Schermuly, and Kauffeld (2015) showed that social competence buffers against the negative effects of faultlines on social loafing behavior in work groups.

#### Summary

Our findings suggest that (aggregated) individual differences are important moderators of relational demography and work group diversity effects. Openness, need for cognition, learning goal orientation, and diversity beliefs were found to promote social integration via positive intergroup contact and enhance performance through information‐elaboration in demographically diverse work groups performing tasks with a strong informational and decision‐making component in particular. Extroversion, self‐monitoring, and positive stereotypes were shown to safeguard against negative affective‐evaluative responses towards demographically dissimilar group members and, in turn, prevent lower social integration. With respect to personality traits, beyond Openness to Experience, research on the moderating effects of the Big Five remains underdeveloped. As Conscientiousness, Agreeableness, and Emotional Stability are also likely to aid individuals and teams in exploiting and coping with demographic differences (because Agreeableness and Emotional Stability may promote positive interpersonal relations, and Conscientiousness may facilitate information‐elaboration), future research should examine whether these factors also moderate workplace diversity effects on social integration, well‐being, and performance. Further, it would be interesting to examine whether need for structure and tolerance of ambiguity moderate the effects of workplace diversity as they might influence how people cope with uncertainty. Recent perspectives on personality development and change (Woods et al., 2013) also invites longitudinal intervention research to examine the interactive effects of demographic diversity with change in individual differences (at individual and workgroup levels) over time on performance and other outcomes. Earlier conceptual work has identified intelligence, general, and task‐related KSAs, communication skills, and individual differences in group members' motivation to work with the group as important moderators of workplace diversity effects based on the notion that they enhance either performance on complex tasks via more information‐elaboration or facilitate social integration (van Knippenberg et al., 2004). We did find some support for the moderating role of social competence, task motivation, and self‐efficacy, but this is clearly an area where more research is needed. Task‐related and team‐related KSAs as well as variables influencing people's prosocial and epistemic motivation seem to be important in this regard.

## Directions for Future Research

Our review confirms earlier findings that main effect approaches are not suited to explain the effects of workplace diversity on social integration, performance, and well‐being‐related variables. In line with the CEM (van Knippenberg et al., 2004), we found across levels that workplace diversity was positively related to performance when the task had strong information‐processing and decision‐making components and when the moderating variables could be associated with eliminating intergroup bias and facilitating information‐elaboration. While there were hardly any studies that examined the effects of organizational diversity on social integration and the effects of workplace diversity (organizational diversity, work group diversity, and relational demography) on well‐being, our findings support the idea put forward by the CEM that moderating variables that eliminate intergroup bias might safeguard against the negative effects of workplace diversity on social integration, performance, and well‐being. As only a few studies actually examined the underlying mechanisms at the individual and organizational levels, our review can of course not prove that the processes identified by the CEM account for the effects of workplace diversity across all levels. Even so, there is strong evidence that the CEM accounts for the effects of workplace diversity at the group level, and we believe that the model has great promise to explain the effects of workplace diversity at the organizational and individual levels.

Our findings also highlight in particular in the area of relational demography that workplace diversity might enfold its effects not only via intergroup bias and information‐elaboration but also through uncertainty reduction (Chattopadhyay et al., 2011; Guillaume et al., 2014). We found that people who are demographically dissimilar experience greater uncertainty about how to interact with dissimilar others and future research might therefore want to examine how this process that has mainly been investigated at the individual level can be integrated with the CEM and unfolds at the group and organizational levels. Echoing earlier findings (Joshi et al., 2011), we encountered few studies that examined the effects of workplace diversity in a multilevel framework, and it would be interesting to see more work that examines how the different types of workplace diversity (relational demography, work group diversity, and organizational diversity) interact with each other and unfold their effects across levels. Our arguments so far point towards a homologous multilevel theory involving parallel relationships between parallel constructs at different levels of analysis (Kozlowski & Klein, 2000). However, this might turn out to be an oversimplification, and the proposed underlying processes might materialize and unfold in different ways at the individual, group, and organizational levels.

**Figure 1:**
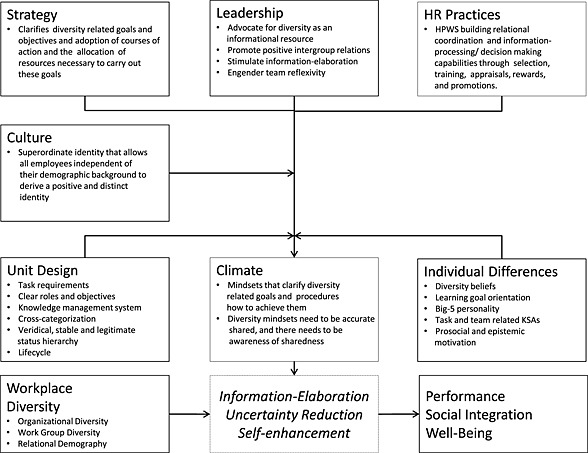
Proposed moderators of workplace diversity effects on social integration, performance, and well‐being‐related variables

Next to advancing research on the different types of moderator variables independently from each other as discussed earlier, our findings also raise interesting questions as to how the different types of moderator variables are interrelated (Figure 1). A better understanding of these interrelationships might help future research to develop an integrative diversity management framework and better explain when workplace diversity yields positive outcomes. In line with previous work (e.g., Avery & McKay, 2010; van Knippenberg et al., 2013; Guillaume et al., 2014), we would expect that climate is likely to be the most proximal moderator of workplace diversity effects as it captures how things are carried out in a work group or organization (Schneider, Ehrhart, & Macey, 2013). We found little support for the idea that generic diversity climate are effective means to manage diversity. Instead, effective diversity climate is likely context specific and might have to clarify diversity‐related goals and procedures how to achieve them and to be most effective might have to be accurate, shared, and there might have to be awareness of sharedness (cf. van Knippenberg et al., 2013).

In line with the authors, we would argue that leadership is likely to play a key role in creating such climates through advocating diversity as an informational resource, stimulating information‐elaboration, promoting positive intergroup contact, and engendering team reflexivity. Strategy is likely to inform diversity‐related goals and procedures and might determine the allocation of resources to carry out these goals. Depending on whether the organization's strategy is to harness diversity as an informational resource or only promote equality and fairness, diversity mind‐sets aimed at facilitating information‐elaboration and/or reducing intergroup bias are likely to ensue. HR practices might also play an important role in developing diversity mind‐sets as recruitment, selection, training, appraisal, reward, and promotion practices might enable and motivate employees to develop and apply relational coordination and information/decision‐making capabilities. Further, the impact of leadership, strategy, and HR practices on diversity climate will likely be exacerbated when they are well aligned.

The effects of leadership, strategy, and HR practices on climate might also be contingent on unit design variables, individual differences, and organizational culture. Facilitating information‐elaboration might be particularly relevant on creative and decision‐making tasks and when diversity is associated with task‐relevant knowledge. Cross‐cutting demographic subgroups, clear roles and objectives, a veridical, stable, and legitimate status hierarchy, and knowledge management systems will likely amplify the positive effects of leadership, strategy, and HR practices on climate as they might facilitate positive intergroup relations and clarify interpersonal conduct. Strategy‐clarifying diversity‐related goals and objectives, leadership advocating diversity as an informational resource, and HR interventions targeting employees' KSAs and motivation might be most effective at the beginning of a task cycle, leadership that facilitates positive intergroup relations and information‐elaboration might be most effective during the midpoint, and interventions targeting learning and reflexivity might be most effective at the endpoint (cf. Hackman & Wageman, 2005). The degree to which people are able and motivated to establish positive intergroup relations and elaborate information likely also depends on their diversity beliefs, personality, task and team‐related KSAs, and prosocial and epistemic motivation. Likewise, culture might moderate the effects. When there is a superordinate identity that allows all employees independent of their background to derive a positive and distinct identity leadership, strategy, and HR practices are likely more effective as there will be buy in from a greater number of people and leaders with dissimilar demographic backgrounds are more likely to be accepted (Haslam et al., 2003; Hornsey & Hogg, 2000a, 2000b).

## Practical Implications

The importance of research influencing practice in the discipline can hardly be greater in any area than in this. Ensuring that we create conditions that enable diversity to be a benefit rather than a hindrance in work communities is vital in human as well as organizational terms. An overriding important practical implication arising from the CEM and the findings of our review is the recognition that demographic differences need to be effectively managed if they are not to lead to lower social integration, performance, and well‐being. Given the theoretical importance of the distinction between social categorization and intergroup bias, it would be wise to raise awareness in diverse organizations of these two processes, and the fact that they are not necessarily overlapping. Where employees clearly understand these two processes and their differential outcomes, it may be more likely that the benefits of diversity will accrue. It is also important to recognize that demographic differences are associated with better performance and higher levels of innovation when associated with task‐relevant knowledge and where there is a clear requirement for growth and team or organizational innovation.

Our findings (Table 1) imply that well‐designed teams and organizations are important to manage diversity effectively. Making certain that teams and groups are created in organizations with clear objectives and roles and making knowledge management systems available is important for ensuring that diversity can be harnessed as an informational resource. Eliminating status differences between demographic subgroups that are not based on merit and building teams and groups in which demographic attributes do not overlap with functional roles is essential to facilitate social integration, performance, and employee well‐being. Beyond diversity training for teams building on the principles of the CEM, we found little evidence that HR practices facilitate positive outcomes of workplace diversity. This is not to say that HR practices are unimportant but that more research is needed to identify those selection, appraisal, rewards, and promotion practices as well as leadership development activities that promote the building of relational coordination and information‐processing/decision‐making capabilities within demographically diverse organizations. Among the studies of individual differences that we reviewed, variables, such as positive attitudes about and beliefs towards diversity, as well as personality‐related variables, such as openness and learning goal orientations, all influence effects of workplace diversity positively. Task and team‐related competencies and motivation were also found to be relevant. These are all obvious targets for employee learning and development to encourage attitudes, beliefs, and working styles and behavior that would promote positive outcomes of workplace diversity.

**Table 1:** Empirically supported moderators of workplace diversity effects on social integration, performance, and well‐being‐related variables.

	Social integration and well‐being	Performance
Strategy		Growth‐oriented strategy (+)
		Innovation strategy (+)
		Downsizing strategy (−)
		Diversity management strategy (+)
		Munificent environments (+)
Unit design	Cross‐categorization (+)	Innovation and creativity tasks (+)
	Faultlines (−)	Decision‐making tasks (+)
	Faultlines*diversity beliefs/openness (+)	Complex tasks when diversity is associated with task‐relevant knowledge and expertise (+)
	Unequal subgroup status*permeable, stable, legitimate status hierarchy (low status: +, high status: −)	Cross‐categorization (+)
		Faultlines (−)
		Faultlines*diversity beliefs/openness (+)
		Unequal subgroup status*positive intergroup relations (+)
	Unequal subgroup status*positive intergroup relations (+)	Autonomy (−)
		Clear roles and shared objectives (+)
	Autonomy (−)	Decision support systems (+)
HR practices	Diversity training*diversity beliefs (negative beliefs/high diversity: +)	Diversity training*diversity beliefs (negative beliefs/high diversity: +)
Leadership	Inclusive (transformational) leadership (+)	Inclusive (transformational) leadership (+)
	Leader openness (+)	LMX (+)
	Leader–follower similarity (+)	LMX differentiation (−)
		Leader openness (+)
		Leader–follower similarity (+)
Climate/culture	Climate for justice (+)	Psychological safety (+)
	Psychological safety (+)	Knowledge sharing/integration norms and mindsets (+)
	Trust (+)	Team climate for innovation (+)
		Political correctness (+)
Individual differences	Extraversion (+)	Openness (+)
	Self‐monitoring (+)	Diversity beliefs (+)
	Openness (+)	Learning goal orientation (+)
	Diversity beliefs (+)	Need for cognition (+)
	Need for cognition (+)	Creative self‐efficacy*KWKW (+)
	Negative stereotypes (−)	Diversity beliefs*task motivation (+)
		Social competence (+)

HR, human resource; KWKW, knowledge of who knows what.

Our findings also highlight the importance of leadership. Leaders, who are participative and inclusive in their approaches, offer inspiring visions, show individualized consideration, and encourage those they lead to engage with their tasks effectively, create the conditions where diversity benefits accrue. This is undermined where leaders show clear biases in relations with those they lead. Equally, the research suggests the value of ensuring that teams, groups, and departments are encouraged to create a climate of equality in relations between team members and to ensure that leaders and authority figures emphasize the value of diversity, civility, and mutual respect. Furthermore, the research on information‐elaboration implies that climates where listening and learning from each other are emphasized are likely to produce work groups and organizations that have positive diversity outcomes. While we found little support for the moderating role of organizational cultures, we would expect that future research will show that organizational cultures in which all employees independent of their demographic background can derive a positive and distinct identity contribute to the creation of diversity environments that ensure the benefits rather than the dysfunctions of diversity accrue.

## References

[job2040-bib-0001] * Ali, M. , Kulik, C. T. , & Metz, I. (2011). The gender diversity‐performance relationship in services and manufacturing organizations. International Journal of Human Resource Management, 22, 1464–1485. doi: 10.1080/09585192.2011.561961

[job2040-bib-0002] Anderson, N. R. , & West, M. A. (1998). Measuring climate for work group innovation: development and validation of the team climate inventory. Journal of Organizational Behavior, 19, 235–258. 10.1002/(SICI)1099-1379(199805)19:3<235::AID-JOB837>3.0.CO;2-C.

[job2040-bib-0003] * Andrevski, G. , Richard, O. C. , Shaw, J. D. , & Ferrier, W. J. (2014). Racial diversity and firm performance: The mediating role of competitive intensity. Journal of Management, 40, 820–844. doi: 10.1177/0149206311424318

[job2040-bib-0004] Avery, D. R. , & McKay, P. F. (2010). Doing diversity right: An empirically based approach to effective diversity management In Hodgkinson G. P., & Ford J. K. (Eds.), International review of industrial and organizational psychology (Vol. 25, pp. 227–252). Oxford, UK: Wiley‐Blackwell.

[job2040-bib-0005] * Avery, D. R. , McKay, P. F. , & Wilson, D. C. (2007). Engaging the aging workforce: The relationship between perceived age similarity, satisfaction with coworkers, and employee engagement. Journal of Applied Psychology, 92, 1542–1556. doi: 10.1037/0021-9010.92.6.1542 18020795 10.1037/0021-9010.92.6.1542

[job2040-bib-0006] * Avery, D. R. , Wang, M. , Volpone, S. D. , & Zhou, L. (2013). Different strokes for different folks: The impact of sex dissimilarity in the empowerment‐performance relationship. Personnel Psychology, 66, 757–784. doi: 10.1111/peps.12032

[job2040-bib-0007] * Bacharach, S. B. , Bamberger, P. A. , & Vashdi, D. (2005). Diversity and homophily at work: Supportive relations among white and African‐American peers. Academy of Management Journal, 48, 619–644. doi: 10.5465/AMJ.2005.17843942

[job2040-bib-0008] * Barkema, H. G. , & Shvyrkov, O. (2007). Does top management team diversity promote or hamper foreign expansion? Strategic Management Journal, 28, 663–680. doi: 10.1002/smj.604

[job2040-bib-0009] * Bezrukova, K. , Spell, C. S. , & Perry, J. L. (2010). Violent splits or healthy divides? Coping with injustice through faultlines. Personnel Psychology, 63, 719–751. doi: 10.1111/j.1744-6570.2010.01185.x

[job2040-bib-0010] * Boerner, S. , Linkohr, M. , & Kiefer, S. (2011). Top management team diversity: Positive in the short run, but negative in the long run? Team Performance Management, 17, 328–353. doi: 10.1108/13527591111182616

[job2040-bib-0011] * Boone, C. , & Hendriks, W. (2009). Top management team diversity and firm performance: Moderators of functional‐background and locus‐of‐control diversity. Management Science, 55, 165–180. doi: 10.1287/mnsc.1080.0899

[job2040-bib-0012] * Brodbeck, F. C. , Guillaume, Y. R. F. , & Lee, N. J. (2011). Ethnic diversity as a multilevel construct: The combined effects of dissimilarity, group diversity, and societal status on learning performance in work groups. Journal of Cross‐Cultural Psychology, 42, 1198–1218. doi: 10.1177/0022022110383314

[job2040-bib-0013] * Bunderson, J. S. (2003). Team member functional background and involvement in management teams: Direct effects and the moderating role of power centralization. Academy of Management Journal, 46, 458–474. doi: 10.2307/30040638

[job2040-bib-0014] * Buyl, T. , Boone, C. , Hendriks, W. , & Matthyssens, P. (2011). Top management team functional diversity and firm performance: The moderating role of CEO characteristics. Journal of Management Studies, 48, 151–177. doi: 10.1111/j.1467-6486.2010.00932.x

[job2040-bib-0015] * Cannella, Jr., A. A. , Park, J.‐H. , & Lee, H.‐U. (2008). Top management team functional background diversity and firm performance: Examining the roles of team member colocation and environmental uncertainty. Academy of Management Journal, 51, 768–784. doi: 10.5465/AMJ.2008.33665310

[job2040-bib-0016] * Carpenter, M. A. (2002). The implications of strategy and social context for the relationship between top management team heterogeneity and firm performance. Strategic Management Journal, 23, 275–284. doi: 10.1002/smj.226

[job2040-bib-0017] * Carpenter, M. A. , & Fredrickson, J. W. (2001). Top management teams, global strategic posture, and the moderating role of uncertainty. Academy of Management Journal, 44, 533–545. doi: 10.2307/3069368

[job2040-bib-0018] Chandler, A. D. (1962). Strategy and structure: Chapters in the history of the American industrial enterprise. Cambridge, MA: The MIT Press.

[job2040-bib-0019] * Chatman, J. A. , & Flynn, F. J. (2001). The influence of demographic heterogeneity on the emergence and consequences of cooperative norms in work teams. Academy of Management Journal, 44, 956–974. doi: 10.2307/3069440

[job2040-bib-0020] * Chatman, J. A. , & O'Reilly, C. A. (2004). Asymmetric reactions to work group sex diversity among men and women. Academy of Management Journal, 47, 193–208. doi: 10.2307/20159572

[job2040-bib-0021] * Chatman, J. A. , Polzer, J. T. , Barsade, S. G. , & Neale, M. A. (1998). Being different yet feeling similar: The influence of demographic composition and organizational culture on work processes and outcomes. Administrative Science Quarterly, 43, 749–780. doi: 10.2307/2393615

[job2040-bib-0022] * Chatman, J. A. , & Spataro, S. E. (2005). Using self‐categorization theory to understand relational demography‐based variations in people's responsiveness to organizational culture. Academy of Management Journal, 48, 321–331. doi: 10.5465/AMJ.2005.16928415

[job2040-bib-0023] * Chattopadhyay, P. (1999). Beyond direct and symmetrical effects: The influence of demographic dissimilarity on organizational citizenship behavior. Academy of Management Journal, 42, 273–287. doi: 10.2307/256919

[job2040-bib-0024] * Chattopadhyay, P. (2003). Can dissimilarity lead to positive outcomes? The influence of open versus closed minds. Journal of Organizational Behavior, 24, 295–312. doi: 10.1002/job.188

[job2040-bib-0025] * Chattopadhyay, P. , Finn, C. , & Ashkanasy, N. M. (2010). Affective responses to professional dissimilarity: A matter of status. Academy of Management Journal, 53, 808–826. doi: 10.5465/AMJ.2010.52814603

[job2040-bib-0026] * Chattopadhyay, P. , George, E. , & Lawrence, S. A. (2004). Why does dissimilarity matter? Exploring self‐categorization, self‐enhancement, and uncertainty reduction. Journal of Applied Psychology, 89, 892–900. doi: 10.1037/0021-9010.89.5.892 15506868 10.1037/0021-9010.89.5.892

[job2040-bib-0027] Chattopadhyay, P. , George, E. , & Ng, C. K. (2011). An uncertainty reduction model of relational demography In Joshi A., Liao H. & Martocchio J. J. (Eds.), Research in personnel and human resource management (Vol. 30, pp. 219–251). Bingley, UK: Emerald Group Publishing Limited.

[job2040-bib-0028] Chattopadhyay, P. , George, E. , & Ng, C. K. (2015). Hearts and minds: Integrating regulatory focus and relational demography to explain responses to dissimilarity. Organizational Psychology Review. Advance online publication. doi: 10.1177/2041386615574540

[job2040-bib-0029] * Chattopadhyay, P. , George, E. , & Shulman, A. D. (2008). The asymmetrical influence of sex dissimilarity in distributive vs. colocated work groups. Organization Science, 19, 581–593. doi: 10.1287/orsc.1070.0324

[job2040-bib-0030] Chattopadhyay, P. , Tluchowska, M. , & George, E. (2004). Identifying the ingroup: A closer look at the influence of demographic dissimilarity on employee identity. Academy of Management Review, 29, 180–202. 10.5465/AMR.2004.12736071.

[job2040-bib-0031] * Chi, N. W. , Lin, S. C. , & Huang, Y. M. (2009). A double‐edged sword? Exploring the curvilinear relationship between organizational tenure diversity and team innovation: The moderating role of team‐oriented HR practices. Group and Organization Management, 34, 698–726. doi: 10.1177/1059601109350985

[job2040-bib-0032] Choi, J. N. (2007). Group composition and employee creative behaviour in a Korean electronics company: Distinct effects of relational demography and group diversity. Journal of Occupational and Organizational Psychology, 80, 213–234. 10.1348/096317906X110250.

[job2040-bib-0033] * Choi, S. (2013). Demographic diversity of managers and employee job satisfaction: Empirical analysis of the federal case. Review of Public Personnel Administration, 33, 275–298. doi: 10.1177/0734371X12453054

[job2040-bib-0034] * Choi, S. , & Rainey, H. G. (2010). Managing diversity in U.S. federal agencies: Effects of diversity and diversity management on employee perceptions of organizational performance. Public Administration Review, 70, 109–121. doi: 10.1111/j.1540-6210.2009.02115.x

[job2040-bib-0035] Cohen, S. G. , & Bailey, D. E. (1997). What makes teams work: Group effectiveness research from the shop floor to the executive suite. Journal of Management, 23, 239–290. 10.1177/014920639702300303.

[job2040-bib-0036] Cox, T. H. (1993). Cultural diversity in organizations: Theory, research and practice. San Francisco, CA: Berrett‐Koehler Publishers.

[job2040-bib-0037] * Cunningham, G. B. (2009). The moderating effect of diversity strategy on the relationship between racial diversity and organizational performance. Journal of Applied Social Psychology, 39, 1445–1460. doi: 10.1111/j.1559-1816.2009.00490.x

[job2040-bib-0038] * Daily, B. , Whatley, A. , Ash, S. R. , & Steiner, R. L. (1996). The effects of a group decision support system on culturally diverse and culturally homogeneous group decision making. Information & Management, 30, 281–289. doi: 10.1016/S0378-7206(96)01062-2

[job2040-bib-0039] Delery, J. E. , & Doty, D. H. (1996). Modes of theorizing in strategic human resource management: Tests of universalistic, contingency, and configurational performance predictions. Academy of Management Journal, 39, 802–835. 10.2307/256713.

[job2040-bib-0040] * Díaz‐García, C. , González‐Moreno, A. , & Sáez‐Martínez, F. J. (2013). Gender diversity within R&D teams: Its impact on radicalness of innovation. Innovation: Management, Policy & Practice, 15, 149–160. doi: 10.5172/impp.2013.15.2.149

[job2040-bib-0041] Dovidio, J. , Gaertner, S. , & Validzic, A. (1998). Intergroup bias: Status, differentiation, and a common in‐group identity. Journal of Personality and Social Psychology, 75, 109–120. 10.1037/0022-3514.75.1.109. 9686453 10.1037//0022-3514.75.1.109

[job2040-bib-0042] * Drach‐Zahavy, A. , & Trogan, R. (2013). Opposites attract or attack? The moderating role of diversity climate in the team diversity‐interpersonal aggression relationship. Journal of Occupational Health Psychology, 18, 449–457. doi: 10.1037/a0033989 24099164 10.1037/a0033989

[job2040-bib-0043] * Dwyer, S. , Richard, O. C. , & Chadwick, K. (2003). Gender diversity in management and firm performance: The influence of growth orientation and organizational culture. Journal of Business Research, 56, 1009–1019. doi: 10.1016/S0148-2963(01)00329-0

[job2040-bib-0044] Elfenbein, H. A. , & O'Reilly, C. A. III (2007). Fitting in: The effects of relational demography and person‐culture fit on group process and performance. Group and Organization Management, 32, 109–142. 10.1177/1059601106286882.

[job2040-bib-0045] * Elvira, M. M. , & Cohen, L. E. (2001). Location matters: A cross‐level analysis of the effects of organizational sex composition on turnover. Academy of Management Journal, 44, 591–605. doi: 10.2307/3069373

[job2040-bib-0046] * Ely, R. J. (2004). A field study of group diversity, participation in diversity education programs, and performance. Journal of Organizational Behavior, 25, 755–780. doi: 10.1002/job.268

[job2040-bib-0047] * Fay, D. , Borrill, C. , Amir, Z. , Haward, R. , & West, M. A. (2006). Getting the most out of multidisciplinary teams: A multi‐sample study of team innovation in health care. Journal of Occupational & Organizational Psychology, 79, 553–567. doi: 10.1348/096317905X72128

[job2040-bib-0048] * Flynn, F. J. , Chatman, J. A. , & Spataro, S. E. (2001). Getting to know you: The influence of personality on impressions and performance of demographically different people in organizations. Administrative Science Quarterly, 46, 414–442. doi: 10.2307/3094870

[job2040-bib-0049] * Gibson, C. B. , & Gibbs, J. L. (2006). Unpacking the concept of virtuality: The effects of geographic dispersion, electronic dependence, dynamic structure, and national diversity on team innovation. Administrative Science Quarterly, 51, 451–495. doi: 10.2189/asqu.51.3.451

[job2040-bib-0050] * Gilson, L. L. , Lim, H. S. , Luciano, M. M. , & Choi, J. N. (2013). Unpacking the cross‐level effects of tenure diversity, explicit knowledge, and knowledge sharing on individual creativity. Journal of Occupational & Organizational Psychology, 86, 203–222. doi: 10.1111/joop.12011

[job2040-bib-0051] * Goldberg, C. B. , Riordan, C. , & Schaffer, B. S. (2010). Does social identity theory underlie relational demography? A test of the moderating effects of uncertainty reduction and status enhancement on similarity effects. Human Relations, 63, 903–926. doi: 10.1177/0018726709347158

[job2040-bib-0052] * Goncalo, J. A. , Chatman, J. A. , Duguid, M. M. , & Kennedy, J. A. (2014). Creativity from constraint? How political correctness influences creativity in mixed‐sex work groups. Retrieved [21/11/2014] from Cornell University, ILR School site: http://digitalcommons.ilr.cornell.edu/articles/910.

[job2040-bib-0053] * Gonzalez, J. A. (2013). Matchmaking: Community and business unit racial/ethnic diversity and business unit performance. International Journal of Human Resource Management, 24, 4063–4081. doi: 10.1080/09585192.2013.792858

[job2040-bib-0054] * Gonzalez, J. A. , & DeNisi, A. S. (2009). Cross‐level effects of demography and diversity climate on organizational attachment and firm effectiveness. Journal of Organizational Behavior, 30, 21–40. doi: 10.1002/job.498

[job2040-bib-0055] * Greer, L. L. , De Hoogh, A. H. B. , Den Hartog, D. N. , & Homan, A. C. (2012). Tainted visions: The effect of visionary leader behaviors and leader categorization tendencies on the financial performance of ethnically diverse teams. Journal of Applied Psychology, 97, 203–213. doi: 10.1037/a0025583 21942407 10.1037/a0025583

[job2040-bib-0056] * Guillaume, Y. R. F. , Brodbeck, F. C. , & Riketta, M. (2012). Surface‐ and deep‐level dissimilarity effects on social integration and individual effectiveness related outcomes in work groups: A meta‐analytic integration. Journal of Occupational and Organizational Psychology, 85, 80–115. doi: 10.1111/j.2044-8325.2010.02005.x

[job2040-bib-0057] Guillaume, Y. R. F. , Dawson, J. F. , Priola, V. , Sacramento, C. A. , Woods, S. A. , Higson, H. E. , … West, M. A. (2014). Managing diversity in organizations: An integrative model and agenda for future research. European Journal of Work and Organizational Psychology, 23, 783–802. doi: 10.1080/1359432X.2013.805485

[job2040-bib-0058] Guillaume, Y. R. F. , Dawson, J. F. , Woods, S. A. , Sacramento, C. A. , & West, M. A. (2013). Getting diversity at work to work: What we know and what we still don't know. Journal of Occupational and Organizational Psychology, 86, 123–141. 10.1111/joop.12009.

[job2040-bib-0059] * Guillaume, Y. R. F. , van Knippenberg, D. , & Brodbeck, F. (2014). Nothing succeeds like moderation: A social self‐regulation perspective on cultural dissimilarity and performance. Academy of Management Journal, 57, 1284–1308. doi: 10.5465/amj.2013.0046

[job2040-bib-0060] Hackman, J. R. , & Wageman, R. (2005). A theory of team coaching. Academy of Management Review, 30, 269–287. 10.5465/AMR.2005.16387885.

[job2040-bib-0061] Hafsi, T. , & Turgut, G. (2013). Boardroom diversity and its effect on social performance: Conceptualization and empirical evidence. Journal of Business Ethics, 112, 463–479. 10.1007/s10551-012-1272-z.

[job2040-bib-0062] Harrison, D. A. , & Klein, K. J. (2007). What's the difference? Diversity constructs as separation, variety, or disparity in organizations. Academy of Management Review, 32, 1199–1228. 10.5465/AMR.2007.26586096.

[job2040-bib-0063] Harrison, D. A. , Price, K. H. , Gavin, J. H. , & Florey, A. T. (2002). Time, teams, and task performance: Changing effects of surface‐ and deep‐level diversity on group functioning. Academy of Management Journal, 45, 1029–1045. 10.2307/3069328.

[job2040-bib-0064] Haslam, S. A. (2004). Psychology in organizations: The social identity approach (2nd edn). London: Sage.

[job2040-bib-0065] Haslam, S. A. , Eggins, R. A. , & Reynolds, K. J. (2003). The ASPIRe model: Actualizing social and personal identity resources to enhance organizational outcomes. Journal of Occupational and Organizational Psychology, 76, 83–113. 10.1348/096317903321208907.

[job2040-bib-0066] * Hoever, I. J. , van Knippenberg, D. , van Ginkel, W. P. , & Barkema, H. G. (2012). Fostering team creativity: Perspective taking as key to unlocking diversity's potential. Journal of Applied Psychology, 97, 982–996. doi: 10.1037/a0029159 22774764 10.1037/a0029159

[job2040-bib-0067] Hogg, M. A. , & Terry, D. I. (2000). Social identity and self‐categorization processes in organizational contexts. Academy of Management Review, 25, 121–140. 10.5465/AMR.2000.2791606.

[job2040-bib-0068] * Homan, A. C. , Buengeler, C. , Eckhoff, R. A. , Van Ginkel, W. P. , & Voelpel, S. C. (2015). The interplay of diversity training and diversity beliefs on team creativity in nationality diverse teams. Journal of Applied Psychology. Advance online publication. doi: 10.1037/apl0000013 10.1037/apl0000013 25688641

[job2040-bib-0069] * Homan, A. C. , & Greer, L. L. (2013). Considering diversity: The positive effects of considerate leadership in diverse teams. Group Processes & Intergroup Relations, 16, 105–125. doi: 10.1177/1368430212437798

[job2040-bib-0070] * Homan, A. C. , Greer, L. L. , Jehn, K. A. , & Koning, L. (2010). Believing shapes seeing: The impact of diversity beliefs on the construal of group composition. Group Processes & Intergroup Relations, 13, 477–493. doi: 10.1177/1368430209350747

[job2040-bib-0071] * Homan, A. C. , Hollenbeck, J. R. , Humphrey, S. E. , Van Knippenberg, D. , Ilgen, D. R. , & Van Kleef, G. A. (2008). Facing differences with an open mind: Openness to experience, salience of intragroup differences, and performance of diverse work groups. Academy of Management Journal, 51, 1204–1222. doi: 10.5465/AMJ.2008.35732995

[job2040-bib-0072] * Homan, A. C. , Van Kleef, G. A. , De Dreu, C. K. W. , & van Knippenberg, D. (2007). Interacting dimensions of diversity: Cross‐categorization and the functioning of diverse work groups. Group Dynamics, 11, 79–94. doi: 10.1037/1089-2699.11.2.79

[job2040-bib-0073] * Homan, A. C. , Van Knippenberg, D. , Van Kleef, G. A. , & De Dreu, C. K. W. (2007). Bridging faultlines by valuing diversity: Diversity beliefs, information elaboration, and performance in diverse work groups. Journal of Applied Psychology, 92, 1189–1199. doi: 10.1037/0021-9010.92.5.1189 17845079 10.1037/0021-9010.92.5.1189

[job2040-bib-0074] Hornsey, M. J. , & Hogg, M. A. (2000a). Assimilation and diversity: An integrative model of subgroup relations. Personality and Social Psychology Review, 4, 143–156. 10.1207/S15327957PSPR0402_03.

[job2040-bib-0075] Hornsey, M. J. , & Hogg, M. A. (2000b). Subgroup relations: A comparison of mutual intergroup differentiation and common ingroup identity models of prejudice reduction. Personality and Social Psychology Bulletin, 26, 242–256. 10.1177/0146167200264010.

[job2040-bib-0076] House R. J., Hanges P. J., Javidan M., Dorfman P. W., & Gupta N. (Eds.). (2004). Culture, leadership, and organizations: The GLOBE study of 62 societies. Thousand Oaks, CA: Sage Publications.

[job2040-bib-0077] Iverson, R. D. , Zatzick, C. D. , & McCrae, M. M. (2008). High‐performance work systems In Barling J., & Cooper C. (Eds.), The SAGE handbook of organizational behavior (pp. 393–410). London: SAGE Publications Ltd.

[job2040-bib-0078] * Jackson, S. E. , & Joshi, A. (2004). Diversity in social context: A multi‐attribute, multilevel analysis of team diversity and sales performance. Journal of Organizational Behavior, 25, 675–702. doi: 10.1002/job.265

[job2040-bib-0079] * Jehn, K. A. , & Bezrukova, K. (2004). A field study of group diversity, workgroup context, and performance. Journal of Organizational Behavior, 25, 703–729. doi: 10.1002/job.266

[job2040-bib-0080] Jehn, K. A. , Northcraft, G. B. , & Neale, M. A. (1999). Why differences make a difference: A field study of diversity, conflict, and performance in workgroups. Administrative Science Quarterly, 44, 741–763. doi: 10.2307/2667054

[job2040-bib-0081] * Joshi, A. , Liao, H. , & Jackson, S. E. (2006). Cross‐level effects of workplace diversity on sales performance and pay. Academy of Management Journal, 49, 459–481. doi: 10.5465/AMJ.2006.21794664

[job2040-bib-0082] Joshi, A. , Liao, H. , & Roh, H. (2011). Bridging domains in workplace demography research: A review and reconceptualization. Journal of Management, 37, 521–552. 10.1177/0149206310372969.

[job2040-bib-0083] * Joshi, A. , & Roh, H. (2009). The role of context in work team diversity research: A meta‐analytic review. Academy of Management Journal, 52, 599–627. doi: 10.5465/AMJ.2009.41331491

[job2040-bib-0084] * Kearney, E. , & Gebert, D. (2009). Managing diversity and enhancing team outcomes: The promise of transformational leadership. Journal of Applied Psychology, 94, 77–89. doi: 10.1037/a0013077 19186897 10.1037/a0013077

[job2040-bib-0085] * Kearney, E. , Gebert, D. , & Voelpel, S. C. (2009). When and how diversity benefits teams: The importance of team members' need for cognition. Academy of Management Journal, 52, 581–598. doi: 10.5465/AMJ.2009.41331431

[job2040-bib-0086] * King, E. B. , Dawson, J. F. , West, M. A. , Gilrane, V. L. , Peddie, C. I. , & Bastin, L. (2011). Why organizational and community diversity matter: Representativeness and the emergence of incivility and organizational performance. Academy of Management Journal, 54, 1103–1118. doi: 10.5465/amj.2010.001

[job2040-bib-0087] * Kirchmeyer, C. (1995). Demographic similarity to the work Group: A longitudinal study of managers at the rarely career stage. Journal of Organizational Behavior, 16, 67–83. doi: 10.1002/job.4030160109

[job2040-bib-0088] * Kirkman, B. L. , Cordery, J. L. , Mathieu, J. , Rosen, B. , & Kukenberger, M. (2013). Global organizational communities of practice: The effects of nationality diversity, psychological safety, and media richness on community performance. Human Relations, 66, 333–362. doi: 10.1177/0018726712464076

[job2040-bib-0089] * Kooij‐de Bode, H. J. M. , van Knippenberg, D. , & van Ginkel, W. P. (2008). Ethnic diversity and distributed information in group decision making: The importance of information elaboration. Group Dynamics, 12, 307–320. doi: 10.1037/1089-2699.12.4.307

[job2040-bib-0157] Kozlowski, S. W. J. , & Klein, K. J. (2000). A multilevel approach to theory and research in organizations: Contextual, temporal, and emergent processes In Klein K. J., & Kozlowski S. W. J. (Eds.), Multilevel theory, research, and methods in organizations: Foundations, extensions, and new directions (pp. 3–90). San Francisco, CA: Jossey‐Bass.

[job2040-bib-0090] Kristof‐Brown, A. L. , Zimmerman, R. D. , & Johnson, E. C. (2005). Consequences of individuals' fit at work: A meta‐analysis of person‐job, person‐organization, person‐group, and person‐supervisor fit. Personnel Psychology, 58, 281–342.

[job2040-bib-0091] * Kunze, F. , & Bruch, H. (2010). Age‐based faultlines and perceived productive energy: The moderation of transformational leadership. Small Group Research, 41, 593–620. doi: 10.1177/1046496410366307

[job2040-bib-0092] Lau, D. C. , & Murnighan, J. K. (1998). Demographic diversity and faultlines: The compositional dynamics of organizational groups. Academy of Management Review, 23, 325–340. 10.5465/AMR.1998.533229.

[job2040-bib-0093] Lau, D. C. , & Murnighan, J. K. (2005). Interactions within groups and subgroups: The effects of demographic faultlines. Academy of Management Journal, 48, 645–659. 10.5465/AMJ.2005.17843943.

[job2040-bib-0094] * Lee, C. , & Farh, J. L. (2004). Joint effects of group efficacy and gender diversity on group cohesion and performance. Journal of Applied Psychology, 53, 136–154. doi: 10.1111/j.1464-0597.2004.00164.x

[job2040-bib-0095] * Leonard, J. S. , Levine, D. I. , & Joshi, A. (2004). Do birds of a feather shop together? The effects on performance of employees' similarity with one another and with customers. Journal of Organizational Behavior, 25, 731–754. doi: 10.1002/job.267

[job2040-bib-0096] * Leslie, L. M. (2014). A status‐based multilevel model of ethnic diversity and work unit performance. Journal of Management. Advance online publication. doi: 10.1177/0149206314535436

[job2040-bib-0097] * Liebermann, S. C. , Wegge, J. , Jungmann, F. , & Schmidt, K.‐H. (2013). Age diversity and individual team member health: The moderating role of age and age stereotypes. Journal of Occupational & Organizational Psychology, 86, 184–202. doi: 10.1111/joop.12016

[job2040-bib-0098] * Meyer, B. , & Schermuly, C. C. (2012). When beliefs are not enough: Examining the interaction of diversity faultlines, task motivation, and diversity beliefs on team performance. European Journal of Work and Organizational Psychology, 21, 456–487. doi: 10.1080/1359432X.2011.560383

[job2040-bib-0099] * Meyer, B. , Schermuly, C. C. , & Kauffeld, S. (2015). That's not my place: The interacting effects of faultlines, subgroup size, and social competence on social loafing behaviour in work groups. European Journal of Work and Organizational Psychology. Advance online publication. doi: 10.1080/1359432X.2014.996554

[job2040-bib-0100] * Mohammed, S. , & Angell, L. C. (2004). Surface‐ and deep‐level diversity in workgroups: Examining the moderating effects of team orientation and team process on relationship conflict. Journal of Organizational Behavior, 25, 1015–1039. doi: 10.1002/job.293

[job2040-bib-0101] * Molleman, E. (2005). Diversity in demographic characteristics, abilities and personality traits: Do faultlines affect team functioning? Group Decision & Negotiation, 14, 173–193. doi: 10.1007/s10726-005-6490-7

[job2040-bib-0102] * Murray, A. I. (1989). Top management group heterogeneity and firm performance. Strategic Management Journal, 10, 125–141. 10.1002/smj.4250100710.

[job2040-bib-0103] * Nakui, T. , Paulus, P. B. , & Van Der Zee, K. I. (2011). The role of attitudes in reactions toward diversity in workgroups. Journal of Applied Social Psychology, 41, 2327–2351. doi: 10.1111/j.1559-1816.2011.00818.x

[job2040-bib-0104] * Naranjo‐Gil, D. , Hartmann, F. , & Maas, V. S. (2008). Top management team heterogeneity, strategic change and operational performance. British Journal of Management, 19, 222–234. doi: 10.1111/j.1467-8551.2007.00545.x

[job2040-bib-0105] * Nederveen Pieterse, A. , van Knippenberg, D. , & van Dierendonck, D. (2013). Cultural diversity and team performance: The role of team member goal orientation. Academy of Management Journal, 56, 782–804. doi: 10.5465/amj.2010.0992

[job2040-bib-0106] * Nishii, L. H. (2013). The benefits of climate for inclusion for gender‐diverse groups. Academy of Management Journal, 56, 1754–1774. doi: 10.5465/amj.2009.0823

[job2040-bib-0107] * Nishii, L. H. , & Mayer, D. M. (2009). Do inclusive leaders help to reduce turnover in diverse groups? The moderating role of leader‐member exchange in the diversity to turnover relationship. Journal of Applied Psychology, 94, 1412–1426. doi: 10.1037/a0017190 19916652 10.1037/a0017190

[job2040-bib-0108] Ostroff, C. , Kinicki, A. J. , & Muhammad, R. S. (2012). Organizational culture and climate In Weiner I. B., Muhammad R. S. & Highhouse S. (Eds.), Handbook of psychology, Vol 12: Industrial and organizational psychology (Vol. 12, pp. 643–676). Hoboken, NJ: John Wiley & Sons.

[job2040-bib-0109] * Paletz, S. B. F. , Peng, K. , Maslach, C. , & Erez, M. (2004). Ethnic composition and its differential impact on group processes in diverse teams. Small Group Research, 35, 128–157. doi: 10.1177/1046496403258793

[job2040-bib-0110] * Pelled, L. H. , Xin, K. R. , & Weiss, A. M. (2001). No es como mí: Relational demography and conflict in a Mexican production facility. Journal of Occupational & Organizational Psychology, 74, 63–84. doi: 10.1348/096317901167235

[job2040-bib-0111] * Peters, L. , & Karren, R. J. (2009). An examination of the roles of trust and functional diversity on virtual team performance ratings. Group & Organization Management, 34, 479–504. doi: 10.1177/1059601107312170

[job2040-bib-0112] Peterson, R. A. , & Brown, S. P. (2005). On the use of beta coefficients in meta‐analysis. Journal of Applied Psychology, 90, 175–181. 10.1037/0021-9010.90.1.175. 15641898 10.1037/0021-9010.90.1.175

[job2040-bib-0113] Pettigrew, T. F. (1998). Intergroup contact theory In Hagan J., & Cook K. S. (Eds.), Annual review of psychology (Vol. 49, pp. 65–85). Palo Alto, CA: Annual Reviews. 10.1146/annurev.psych.49.1.65 15012467

[job2040-bib-0114] Randel, A. E. (2002). Identity salience: A moderator of the relationship between group gender composition and work group conflict. Journal of Organizational Behavior, 23, 749–766. 10.2307/4093652.

[job2040-bib-0115] Randel, A. E. , & Jaussi, K. S. (2003). Functional background identity, diversity, and individual performance in cross‐functional teams. Academy of Management Journal, 46, 763–774. doi: 10.2307/30040667

[job2040-bib-0116] Rast, D. E. III , Gaffney, A. M. , Hogg, M. A. , & Crisp, R. J. (2012). Leadership under uncertainty: When leaders who are non‐prototypical group members can gain support. Journal of Experimental Social Psychology, 48, 646–653. 10.1016/j.jesp.2011.12.013.

[job2040-bib-0117] * Richard, O. C. (2000). Racial diversity, business strategy, and firm performance: A resource‐based view. Academy of Management Journal, 43, 164–177. doi: 10.2307/1556374

[job2040-bib-0118] * Richard, O. C. , Barnett, T. , Dwyer, S. , & Chadwick, K. (2004). Cultural diversity in management, firm performance, and the moderating role of entrepreneurial orientation dimensions. Academy of Management Journal, 47, 255–266. doi: 10.2307/20159576

[job2040-bib-0119] * Richard, O. C. , Ford, D. , & Ismail, K. (2006). Exploring the performance effects of visible attribute diversity: The moderating role of span of control and organizational life cycle. International Journal of Human Resource Management, 17, 2091–2109. doi: 10.1080/09585190601000246

[job2040-bib-0120] * Richard, O. C. , McMillan, A. , Dwyer, S. , & Chadwick, K. (2003). Employing an innovation strategy in racially diverse workforces: Effects on firm performance. Group and Organization Management, 28, 107–126. doi: 10.1177/1059601102250022

[job2040-bib-0121] * Richard, O. C. , Murthi, B. P. S. , & Ismail, K. (2007). The impact of racial diversity on intermediate and long‐term performance: The moderating role of environmental context. Strategic Management Journal, 28, 1213–1233. doi: 10.1002/smj.633

[job2040-bib-0122] * Richard, O. C. , & Shelor, R. M. (2002). Linking top management team age heterogeneity to firm performance: Juxtaposing two mid‐range theories. International Journal of Human Resource Management, 13, 958–974. doi: 10.1080/09585190210134309

[job2040-bib-0123] * Richter, A. W. , van Knippenberg, D. , Hirst, G. , & Baer, M. (2012). Creative self‐efficacy and individual creativity in team contexts: Cross‐level interactions with team informational resources. Journal of Applied Psychology, 97, 1282–1290. doi: 10.1037/a0029359 22800186 10.1037/a0029359

[job2040-bib-0124] * Rico, R. , Molleman, E. , Sánchez‐Manzanares, M. , & Van der Vegt, G. S. (2007). The effects of diversity faultlines and team task autonomy on decision quality and social integration. Journal of Management, 33, 111–132. doi: 10.1177/0149206306295307

[job2040-bib-0125] * Sacco, J. M. , & Schmitt, N. (2005). A dynamic multilevel model of demographic diversity and misfit effects. Journal of Applied Psychology, 90, 203–231. doi: 10.1037/0021-9010.90.2.203 15769233 10.1037/0021-9010.90.2.203

[job2040-bib-0126] * Schippers, M. C. , Den Hartog, D. N. , Koopman, P. L. , & Wienk, J. A. (2003). Diversity and team outcomes: The moderating effects of outcome interdependence and group longevity and the mediating effect of reflexivity. Journal of Organizational Behavior, 24, 779–802. doi: 10.1002/job.220

[job2040-bib-0127] Schneider, B. , & Barbera, K. M. (2014). Introduction: The Oxford handbook of organizational climate and culture In Schneider B., & Barbera K. M. (Eds.), The Oxford handbook of organizational climate and culture (pp. 3–20). Oxford, UK: Oxford University Press.

[job2040-bib-0128] Schneider, B. , Ehrhart, K. H. , & Macey, W. H. (2013). Organizational climate and culture In Fiske S. T., Schacter D. L., & Taylor S. E. (Eds.), Annual review of psychology (Vol. 64, pp. 361–388). Palo Alto, CA: Annual Reviews. 10.1146/annurev-psych-113011-143809 22856467

[job2040-bib-0129] * Seong, J. Y. , & Hong, D. S. (2013). Gender diversity: How can we facilitate its positive effects on teams? Social Behavior and Personality, 41, 497–507. doi: 10.2224/sbp.2013.41.3.497

[job2040-bib-0130] * Shin, S. J. , & Zhou, J. (2007). When is educational specialization heterogeneity related to creativity in research and development teams? Transformational leadership as a moderator. Journal of Applied Psychology, 92, 1709–1721. doi: 10.1037/0021-9010.92.6.1709 18020807 10.1037/0021-9010.92.6.1709

[job2040-bib-0131] * Simons, T. (1995). Top management team consensus, heterogeneity, and debate as contingent predictors of company performance: The complimentarity of group structure and process. *Academy of Management Best Papers Proceedings*, 62–66. doi: 10.5465/AMBPP.1995.17536282

[job2040-bib-0132] * Somech, A. (2006). The effects of leadership style and team process on performance and innovation in functionally heterogeneous teams. Journal of Management, 32, 132–157. doi: 10.1177/0149206305277799

[job2040-bib-0133] * Soojin, K. , Keunyoung, S. , Seokho, K. , & Sungzoon, C. (2013). Organizational tenure diversity as predictors of combat performance in ROK Army. Military Psychology, 25, 345–353. doi: 10.1037/mi10000004

[job2040-bib-0134] * Stewart, M. M. , & Johnson, O. E. (2009). Leader‐member exchange as a moderator of the relationship between work group diversity and team performance. Group & Organization Management, 34, 507–535. doi: 10.1177/1059601108331220

[job2040-bib-0135] Tajfel, H. , & Turner, J. (1986). The social identity of intergroup behaviour In Worchel W. A. S. (Ed.), Psychology and intergroup relations. Chicago: Nelson‐Hall.

[job2040-bib-0136] * Thatcher, S. M. B. , & Patel, P. C. (2011). Demographic faultlines: A meta‐analysis of the literature. Journal of Applied Psychology, 96, 1119–1139. doi: 10.1037/a0024167 21688882 10.1037/a0024167

[job2040-bib-0137] * Troester, C. , & van Knippenberg, D. (2012). Leader openness, nationality dissimilarity, and voice in multinational management teams. Journal of International Business Studies, 43, 591–613. doi: 10.1057/jibs.2012.15

[job2040-bib-0138] * Tsui, A. S. , Egan, T. D. , & O'Reilly, C. A. (1992). Being different: Relational demography and organizational attachment. Administrative Science Quarterly, 37, 549–579. doi: 10.2307/2393472

[job2040-bib-0139] Tsui, A. S. , & Gutek, B. (1999). Demographic differences in organizations: Current research and future directions. Lanham, Maryland: Lexington Books.

[job2040-bib-0140] * Tuggle, C. S. , Schnatterly, K. , & Johnson, R. A. (2010). Attention patterns in the boardroom: How board composition and processes affect discussion of entrepreneurial issues. Academy of Management Journal, 53, 550–571. 10.5465/AMJ.2010.51468687.

[job2040-bib-0141] Turner, J. C. , Hogg, M. A. , Oakes, P. J. , Reicher, S. D. , & Wetherell, M. S. (1987). Rediscovering the social group: A self‐categorization theory. Oxford, UK: Blackwell Publishers.

[job2040-bib-0142] Van Der Vegt, G. S. , & Bunderson, J. S. (2005). Learning and performance in multidisciplinary teams: The importance of collective team identification. Academy of Management Journal, 48, 532–547. doi: 10.5465/AMJ.2005.17407918

[job2040-bib-0143] * van Dick, R. , van Knippenberg, D. , Hägele, S. , Guillaume, Y. R. F. , & Brodbeck, F. C. (2008). Group diversity and group identification: The moderating role of diversity beliefs. Human Relations, 61, 1463–1492. doi: 10.1177/0018726708095711

[job2040-bib-0144] * van Dijk, H. , & van Engen, M. L. (2013). A status perspective on the consequences of work group diversity. Journal of Occupational and Organizational Psychology, 86, 223–241. doi: 10.1111/joop.12014

[job2040-bib-0145] * van Dijk, H. , van Engen, M. L. , & van Knippenberg, D. (2012). Defying conventional wisdom: A meta‐analytical examination of the differences between demographic and job‐related diversity relationships with performance. Organizational Behavior and Human Decision Processes, 119, 38–53. doi: 10.1016/j.obhdp.2012.06.003

[job2040-bib-0146] * van Knippenberg, D. , Dawson, J. F. , West, M. A. , & Homan, A. C. (2011). Diversity faultlines, shared objectives, and top management team performance. Human Relations, 64, 307–336. doi: 10.1177/0018726710378384

[job2040-bib-0147] van Knippenberg, D. , De Dreu, C. K. W. , & Homan, A. C. (2004). Work group diversity and group performance: An integrative model and research agenda. Journal of Applied Psychology, 89, 1008–1022. 10.1037/0021-9010.89.6.1008. 15584838 10.1037/0021-9010.89.6.1008

[job2040-bib-0148] * van Knippenberg, D. , Haslam, S. A. , & Platow, M. J. (2007). Unity through diversity: Value‐in‐diversity beliefs, work group diversity, and group identification. Group Dynamics, 11, 207–222. doi: 10.1037/1089-2699.11.3.207

[job2040-bib-0149] * van Knippenberg, D. , & Schippers, M. C. (2007). Work group diversity In Fiske S. T., Kazdin D. L., & Schacter D. L. (Eds.), Annual review of psychology (Vol. 58, pp. 515–541). Palo Alto, CA: Annual Reviews. 10.1146/annurev.psych.58.110405.085546 16903805

[job2040-bib-0150] van Knippenberg, D. , & Sitkin, S. B. (2013). A critical assessment of charismatic‐transformational leadership research: Back to the drawing board? Academy of Management Annals, 7, 1–60. 10.1080/19416520.2013.759433.

[job2040-bib-0151] van Knippenberg, D. , Van Ginkel, W. P. , & Homan, A. C. (2013). Diversity mindsets and the performance of diverse teams. Organizational Behavior and Human Decision Processes, 121, 183–193. 10.1016/j.obhdp.2013.03.003.

[job2040-bib-0152] * Wegge, J. , Roth, C. , Kanfer, R. , Neubach, B. , & Schmidt, K.‐H. (2008). Age and gender diversity as determinants of performance and health in a public organization: The role of task complexity and group size. Journal of Applied Psychology, 93, 1301–1313. doi: 10.1037/a0012680 19025249 10.1037/a0012680

[job2040-bib-0153] Williams, K. Y. , & O'Reilly, C. A. (1998). Demography and diversity in organizations: A review of 40 years of research In Staw B. M., & Sutton R. I. (Eds.), Research in organizational behavior, 20 (Vol. 20, pp. 77–140). New York: Elsevier/JAI.

[job2040-bib-0154] Woods, S. A. , Lievens, F. , De Fruyt, F. , & Wille, B. (2013). Personality across working life: The longitudinal and reciprocal influences of personality on work. Journal of Organizational Behavior, 34, S7–S25. 10.1002/job.1863.

[job2040-bib-0155] * Yang, Y. , & Konrad, A. M. (2011). Diversity and organizational innovation: The role of employee involvement. Journal of Organizational Behavior, 32, 1062–1083. doi: 10.1002/job.724

[job2040-bib-0156] * Zoogah, D. B. , Vora, D. , Richard, O. , & Peng, M. W. (2011). Strategic alliance team diversity, coordination, and effectiveness. International Journal of Human Resource Management, 22, 510–529. doi: 10.1080/09585192.2011.543629

